# Explainable Artificial Intelligence in Rotating Machinery Fault Diagnosis: A Comprehensive Review and Emerging Trends

**DOI:** 10.3390/s26175379

**Published:** 2026-08-25

**Authors:** Shengnan Tang, Zengyu Ren, Leiqi Zheng, Jiaming Wang

**Affiliations:** 1 School of Mechanical Engineering, Jiangsu University, Zhenjiang 212013, China; 2 State Key Laboratory of Mechanical Transmission for Advanced Equipment, Chongqing University, Chongqing 400044, China; 3 Leo Group Co., Ltd., Wenling 317500, China

**Keywords:** rotating machinery, intelligent fault diagnosis, ante hoc explainability, post hoc explainability, deep learning

## Abstract

**Highlights:**

This review systematically surveys industrial explainable AI (XAI) techniques for rotating machinery fault diagnosis, with a balanced coverage of ante-hoc and post-hoc interpretable methods. It identifies critical research challenges and real-world deployment bottlenecks, and outlines future prospects for constructing robust, explainable diagnostic frameworks.

**What are the main findings?**

- Industrial explainability requires transparency, comprehensibility, and credibility, with explanations aligned with physical fault mechanisms.
- Ante hoc and post hoc methods are complementary, but industrial deployment remains limited by inadequate benchmarks, robustness, and actionable outputs.

**What are the implications of the main findings?**

- Standardized evaluation frameworks and lightweight, robust explainable models are needed for complex industrial conditions.
- Integrating physical knowledge, causal inference, and digital twins can support trustworthy diagnosis and actionable maintenance decisions.

**Abstract:**

Rotating machinery is essential to energy, aerospace, manufacturing, and other safety-critical industries. Although deep-learning-based fault diagnosis has achieved high accuracy, its black-box nature limits trust, verification, and industrial deployment. This review examines explainable artificial intelligence for rotating machinery fault diagnosis and classifies existing methods into ante hoc and post hoc approaches according to their integration with model architectures. Their physical interpretability, applicable fault scenarios, explanation quality, computational overhead, robustness, and edge-deployment potential are critically compared. Quantitative criteria, including fidelity, stability, robustness, localization, and physical consistency are discussed to support objective evaluation of explanations. The review further highlights the gap between laboratory validation and industrial operation, particularly under sensor degradation, electromagnetic interference, variable working conditions, limited computing resources, and scarce fault data. It also discusses how model-relative explanations can be mapped to calibrated vibration quantities, fault-characteristic frequencies, industrial diagnostic standards, and actionable maintenance decisions. The distinction between correlation-based attribution and causal root-cause analysis is clarified, together with the role of digital twins and human-in-the-loop decision support. Finally, future research priorities are identified in standardized benchmarking, robust lightweight models, causal reasoning, and human-centered industrial deployment.

## 1. Introduction

Rotating machinery is widely regarded as the power heart of complete sets of industrial equipment. The operating condition of its key components is closely linked to production safety. Undetected faults in these components may trigger unplanned shutdowns and even catastrophic safety accidents [1,2,3]. Therefore, condition monitoring and Fault Diagnosis (FD) for rotating machinery have long been a key research directions in industrial Operation and Maintenance (O&M). Traditional diagnosis methods are typically characterized by offline analysis and post-event tracing, which fail to achieve high-precision online early warning when processing massive real-time data [4]. In complex scenarios, manually designed fault features have insufficient sensitivity, and the generalization ability of diagnosis models can hardly meet the requirements [5]. The widespread adoption of Industrial Internet of Things (IIoT) has brought massive, high-dimensional, multi-source real-time monitoring data, making the traditional analysis mode barely able to meet the industrial online diagnosis and advanced early warning [6].

Driven by the rapid development of DL technologies, a series of models, typically including Convolutional Neural Networks (CNNs), Recurrent Neural Networks (RNNs), and the Transformer architecture, have been increasingly applied to rotating machinery FD [7,8,9]. With superior nonlinear fitting capacity, automatic feature extraction ability, and end-to-end learning merits, these methods have shown significant advantages over traditional ones. In particular, DL models reduce the difficulty of manual feature engineering in incipient weak fault detection and compound FD tasks. When integrated with transfer learning, they can also address cross-domain diagnosis tasks under variable working conditions, overcoming the inherent limitations of traditional methods. With effective improvement in both diagnostic accuracy and automation level, such models have gradually become a core technical support for intelligent O&M systems [10].

However, the large-scale industrial application of DL in rotating machinery Intelligent Fault Diagnosis (IFD) still faces multiple constraints. In addition to core engineering bottlenecks including the scarcity of fault samples, poor adaptability to wide-range variable operating conditions, and insufficient generalization ability under strong noise [11,12,13]. The trustworthiness issues caused by the inherent “black-box” nature of the model are one of the key bottlenecks hindering its deployment in high-safety-critical scenarios [14]. In the feature learning and decision reasoning driven by the model’s multi-layer nonlinear transformation, the screening logic of critical features and the formation rationale of final diagnostic results can hardly be explained or quantitatively characterized. This input–output transparency gap makes the models prone to causing a lack of human–machine trust, and also poses barriers to the reliability auditing and certification of equipment O&M systems in high-safety-critical sectors [15]. In the on-site O&M process, apart from confirming whether the equipment is faulty and the specific fault category, engineers usually expect to further understand what signs the model relies on to make the above judgments, and whether the basis of these judgments is consistent with the physical mechanism of the fault itself.

If the diagnostic results lack explainability, the first problem lies in the level of human–machine trust. Meanwhile, in industries with extremely high requirements for equipment safety and reliability, such as aviation, nuclear power, and petrochemicals, O&M systems must meet the regulatory requirements for full life-cycle fault traceability, while unexplainable black-box models fail to comply with such strict regulatory specifications. Furthermore, when the models produce false positives or false negatives in practical applications, the lack of explainability also poses challenges to tracing the root causes of errors, making it difficult to implement targeted model optimization and carry out cross-validation aligned with fault mechanisms [16].

To break through the aforementioned bottlenecks, the cross-integration of Explainable Artificial Intelligence (XAI) technology with rotating machinery IFD has emerged as a leading research hotspot. Existing studies have centered on the two universal frameworks, namely ante hoc explainability and post hoc explainability. A system of theoretical achievements and methodologies has been formed by integrating the fault mechanism of rotating machinery and the industrial requirements [17]. Ante hoc explainability methods are primarily realized through the integration of embedded physical mechanisms of failure, whereas post hoc explainability methods focus on the technical direction of reverse tracing of model decision logic. Notably, explainable diagnostic techniques for industrial scenarios face unique requirements that distinguish them from laboratory research. Furthermore, they need to adapt to stringent industrial operating conditions [18].

**Figure 1:**
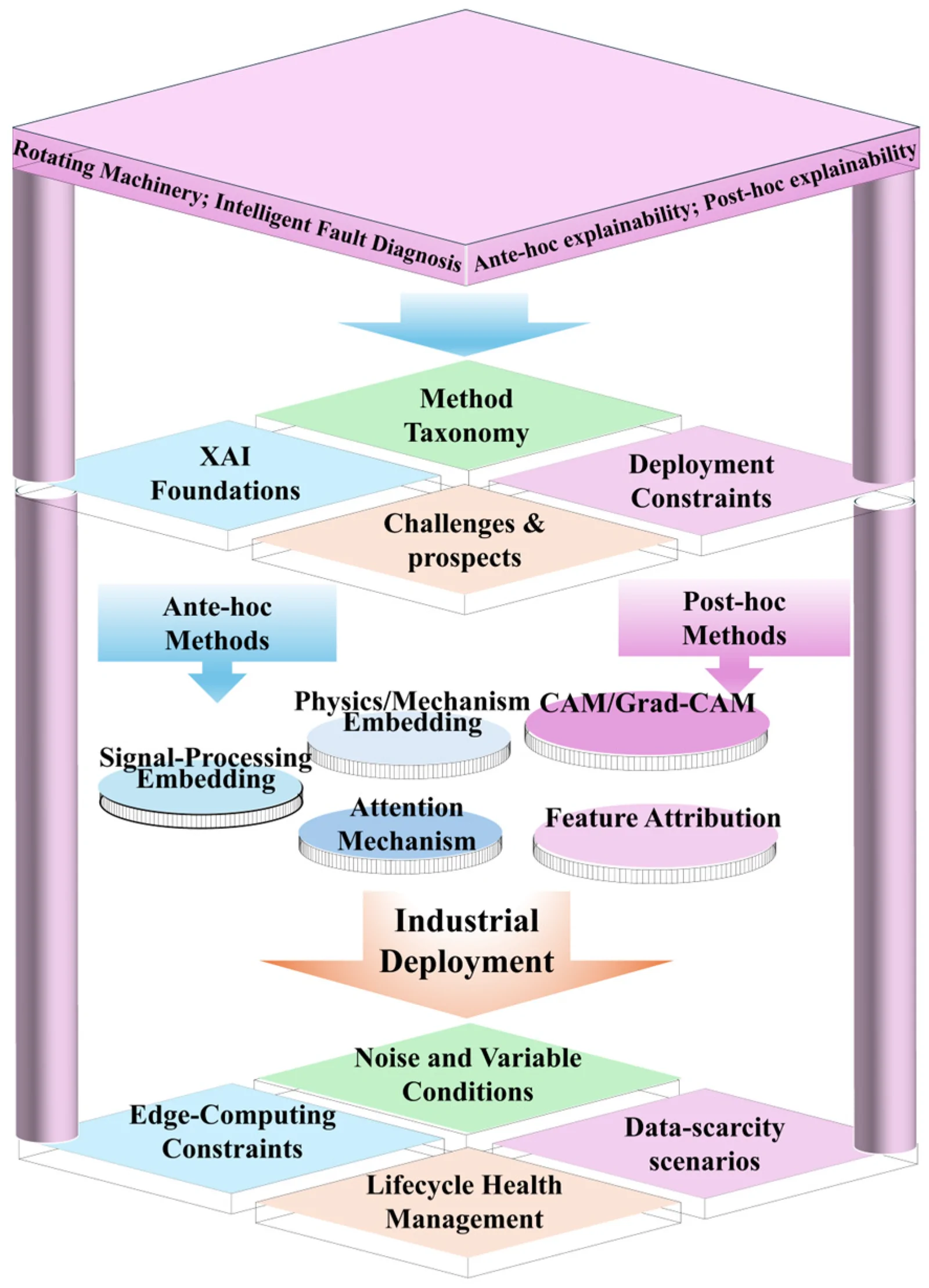
Structural framework diagram of the review.

This paper focuses on the industrial deployment of IFD for rotating machinery and systematically reviews the research progress and technical framework of explainability methods. First, it defines the core connotation and classification of explainability in rotating machinery FD. Second, it elaborates on the technical pathways, innovative achievements, and application boundaries of ante hoc explainability and post hoc explainability. It then concentrates on the key pain points in industrial settings. Finally, it summarizes the core challenges facing current research and discusses future development directions, aiming to offer theoretical references and technical guidance for building trustworthy, usable, and efficient industrial-grade explainable intelligent FD systems. Figure 1 illustrates the structural framework.

## 2. Literature Search and Review Organization

This comprehensive review adopted a structured literature-search and thematic-synthesis procedure to improve the transparency and coverage of the reviewed literature. Relevant PRISMA 2020 reporting items were used to guide the reporting of information sources, search terms, and literature handling [19]; however, the review is not presented as a fully PRISMA-compliant systematic review. The following subsections describe the review scope, search strategy, literature relevance criteria, and thematic synthesis procedure.

### 2.1. Review Scope

This review focuses on explainable artificial intelligence methods for fault diagnosis of bearings, gearboxes, gears, rotors, motors, hydraulic pumps, turbines, and related rotating machinery. The diagnostic tasks considered include fault detection, classification, localization, and severity assessment. Studies dealing exclusively with Remaining Useful Life (RUL) prediction were not treated as core diagnostic evidence, although selected RUL studies were considered when discussing emerging trends and future industrial applications.

Both ante hoc and post hoc explainability were covered. Ante hoc approaches incorporate interpretable signal-processing operations, physical knowledge, transparent structures, or explicit constraints into model development, whereas post hoc approaches explain the predictions of an already trained diagnostic model. The reviewed literature was analyzed in terms of explanation mechanism, diagnostic application, explanation quality, computational overhead, robustness under changing operating conditions, and industrial deployment potential.

### 2.2. Information Sources and Search Strategy

A structured literature search was conducted in the Web of Science Core Collection, IEEE Xplore, and ScienceDirect. These databases were selected because of their coverage of mechanical engineering, signal processing, artificial intelligence, condition monitoring, and industrial fault diagnosis. The search covered English-language publications indexed from database inception to 1 June 2026. Peer-reviewed research and review articles were considered, whereas conference papers, dissertations, patents, books, and other non-peer-reviewed publications were excluded.

The search strategy combined three groups of terms representing the machinery type, diagnostic task, and explainability method. Terms within each group were connected using “OR”, and the three groups were combined using “AND”. The general search expression was:

(“rotating machin*” OR bearing* OR gearbox* OR gear* OR rotor* OR motor* OR “hydraulic pump*”) AND (“fault diagnos*” OR “fault detect*” OR “fault classif*” OR “fault localization” OR “fault severit*”) AND (“explainable artificial intelligence” OR “interpretable artificial intelligence” OR XAI OR explainab* OR interpretab* OR “feature attribution” OR “class activation mapping” OR CAM OR “Grad-CAM” OR SHAP OR LIME OR “integrated gradients” OR “physics-informed” OR “knowledge-informed” OR “mechanism-guided” OR “algorithm unrolling”).

The searches targeted titles, abstracts, and keywords whenever supported by the database. The corresponding topic fields were used in Web of Science, title, abstract, and metadata fields in IEEE Xplore, and title, abstract, and author-specified keyword fields in ScienceDirect. Query syntax was adjusted to the interface and wildcard conventions of each database without changing the three principal concept groups. The database-specific settings are summarized in Table 1.

Studies focusing exclusively on Remaining Useful Life (RUL) prediction were not included in the primary evidence corpus because the central scope of this review is fault detection and diagnosis. Nevertheless, selected explainable RUL studies were consulted in the discussion of emerging trends and future industrial applications.

### 2.3. Eligibility Criteria

Literature relevance was assessed according to the criteria summarized in Table 2. Publications retained for detailed analysis were required to address a clearly defined rotating machinery fault-diagnosis task and to explicitly develop, apply, or evaluate an interpretable model or XAI method. Black-box diagnostic studies without explanation evidence and studies unrelated to rotating machinery diagnosis were not retained.

Potentially relevant publications were first identified from their titles, abstracts, and keywords. Full texts were then consulted when relevance could not be determined from the bibliographic information alone. Duplicate publications were checked manually by comparing titles, authors, publication years, and Digital Object Identifiers (DOIs). When several publications reported substantially the same method or experiment, the most complete version was retained. Review articles were used mainly to establish the theoretical context and identify relevant original studies rather than as substitutes for primary evidence.

### 2.4. Literature Organization and Narrative Synthesis

The full-text PDF files were manually collected and summarized in a Microsoft Word-based literature archive. For each publication, the summary recorded the research objective, machinery and fault type, sensor modality, data source, operating conditions, signal-processing method, diagnostic model, explanation approach, principal findings, reported limitations, and potential industrial relevance. Information not explicitly reported in the original publication was recorded as “not reported” and was not inferred.

The retained literature was organized into thematic categories covering signal-processing-based models, physics- and knowledge-informed models, attention-based approaches, CAM-based visualization, feature-attribution methods, explanation-quality evaluation, and industrial deployment. A publication could be assigned to more than one category when it addressed multiple methodological or application dimensions.

**Figure 2:**
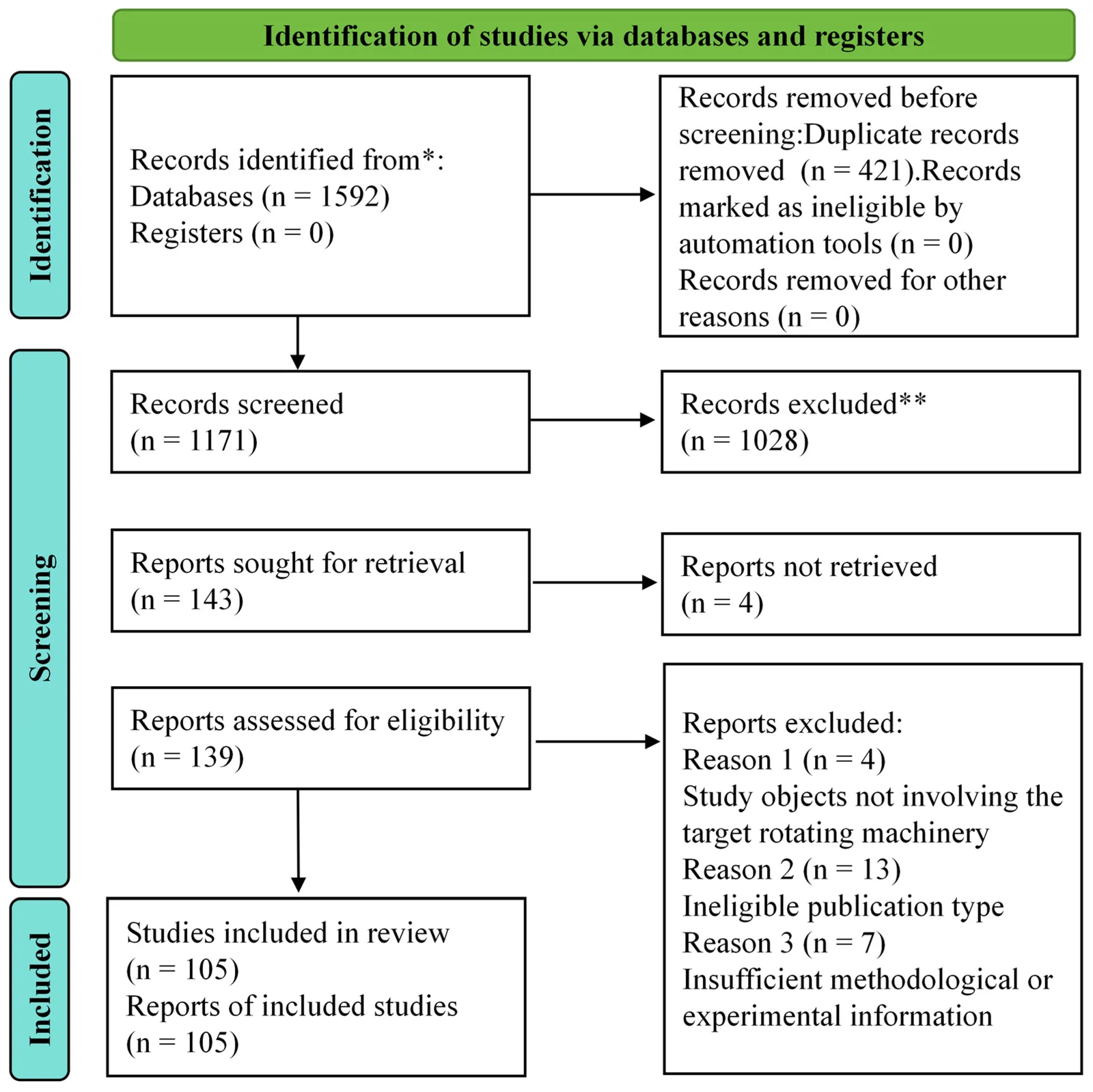
PRISMA-based flow diagram of the literature search and screening process used to establish the original review corpus. Note: * Records identified from each database searched (Table 1). ** No automation tools were used; all records excluded at the screening stage were removed manually.

Because the reviewed studies differ substantially in machinery type, dataset, fault condition, diagnostic task, model architecture, and explanation method, statistical meta-analysis was not considered appropriate. The evidence was therefore synthesized narratively through method-level comparison, representative applications, reported advantages and limitations, computational requirements, robustness, and industrial deployment readiness. Figure 2 provides a descriptive overview of the literature retrieval, relevance assessment, and thematic organization procedure.

## 3. Overview of Explainable Methods

This section establishes an overarching theoretical framework for the entire paper. Specifically, it clarifies the core definition and evaluation dimensions of explainability in industrial scenarios of rotating machinery, formulates the classification system of explainable methods, and identifies the core scenario constraints that hinder the industrial deployment of laboratory-developed methods. This framework thereby lays a rigorous theoretical foundation for the method review and industrial deployment-oriented research.

### 3.1. Fundamentals and Definition of Explainability in Rotating Machinery Fault Diagnosis

Artificial intelligence-based fault diagnosis establishes a mapping between measured machine signals and diagnostic outputs. In a conventional machine-learning pipeline, vibration signals are first transformed into manually designed statistical, spectral, or time-frequency features, which are then used by classifiers such as support vector machines, decision trees, or ensemble models. Deep-learning models perform feature extraction and classification jointly through multiple nonlinear layers, providing stronger representation capacity but reducing the transparency of the decision process. For rotating machinery, the input may include vibration, acoustic-emission, motor-current, temperature, or pressure signals, while the output may correspond to fault detection, fault classification, fault localization, or severity assessment [20,21].

Explainable artificial intelligence aims to reveal why and how an AI model produces a particular diagnostic result. In this review, explainability is distinguished from diagnostic accuracy: accuracy measures whether the prediction is correct, whereas explainability concerns the traceability, comprehensibility, and credibility of the evidence supporting that prediction. Ante hoc methods incorporate interpretable structures, physical constraints, or signal-processing operators into the model before or during training. Post hoc methods generate explanations after a black-box model has been trained, without necessarily changing its inference architecture. The resulting explanations should be understood as model-relative evidence of feature reliance and should not automatically be interpreted as causal proof or calibrated engineering measurements [22,23].

Explainability is the core capability of an AI model to present its internal feature-learning mechanisms and decision-making reasoning logic in a human-comprehensible form [24]. In view of the industrial scenario attributes of rotating machinery FD, this paper decomposes the core connotation of explainability into three progressively hierarchical core dimensions: transparency, comprehensibility, and credibility, which collectively form the fundamental evaluation criteria for explainability in industrial scenarios.

Transparency is a core non-functional requirement of intelligent models in IFD for rotating machinery. It requires that the entire workflow of the model, from vibration signal input to fault decision output, has the inherent properties of observable structure, decomposable logic, and traceable decision-making. Explainability is the core technical means to achieve system transparency and address the black-box problem of DL, as well as the core prerequisite for building the trust of O&M personnel in diagnostic models. This logic is highly consistent with the core research consensus on transparency and explainability in the fields of AI ethics and systems engineering [25]. In FD scenarios, the core requirement for high transparency is that each computational step of the model can be mapped to explicit mathematical or signal processing logic, which fundamentally avoids issues such as uncontrollable decision-making and spurious correlation learning caused by multi-layer nonlinear transformations in black-box models. For example, shallow models such as linear classifiers and decision trees have a fully transparent reasoning structure, while the internal feature representations of traditional deep neural networks are difficult to intuitively map to physical meanings, resulting in a significant lack of transparency [26].

For FD explainability oriented to industrial deployment, the core requirement lies in domain comprehensibility. Unlike explanation outputs targeting general audiences in the general AI field, the core of comprehensibility in rotating machinery FD is that explanation results must be strongly coupled with the physical fault mechanisms of core components, and directly correspond to domain-recognized physical quantities including fault characteristic frequencies, modulation sideband laws, and time-domain impulse characteristics, rather than abstract numerical features or statistical correlations with no physical significance. Only explanation results with domain comprehensibility can assist on-site O&M personnel in accurately judging the rationality of model decisions, and truly realize the engineering deployment value of explainable methods [27].

**Figure 3:**
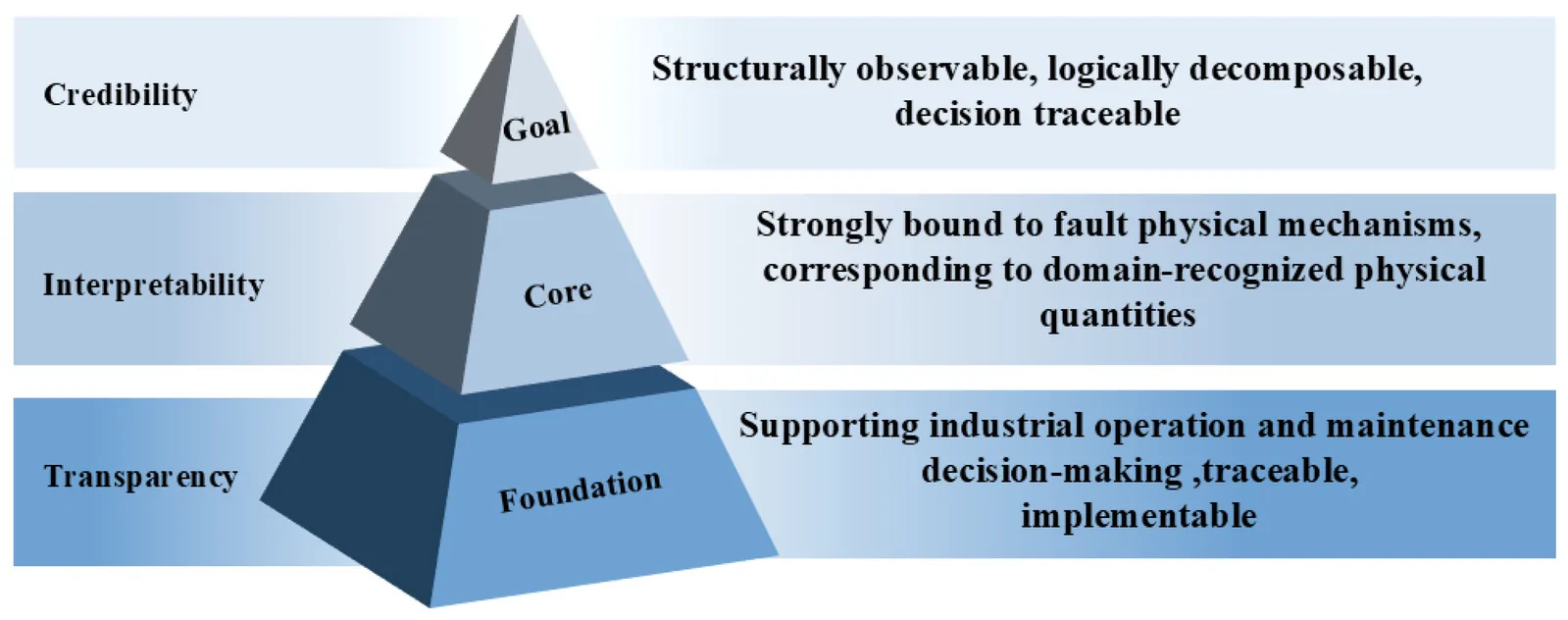
Three-dimensional core connotation progressive relationship diagram of explainability.

In industrial scenarios, the credibility of IFD models is the ultimate goal of explainable methods. Its core is to enable explanation results to support industrial O&M decision-making, which can be evaluated from three core dimensions: the authenticity of the decision-making basis, the traceability of the root causes of misjudgment, and the implementability of O&M support [28]. The aforementioned three dimensions of transparency, comprehensibility, and credibility constitute a progressively hierarchical core system of explainability for rotating machinery FD in industrial scenarios, whose hierarchical relationships and core connotations are illustrated in Figure 3.

### 3.2. Classification System of Explainable Methods

Based on the core connotation and evaluation system of explainability for industrial rotating machinery FD established in the previous section, combined with the technical characteristics of rotating machinery FD and industrial deployment requirements, this paper takes the timing of explanation generation and the degree of coupling with the model architecture as the core criteria, and divides the existing explainable methods into two main categories: ante hoc explainable methods and post hoc explainable methods, which covers the mainstream research directions [29].

Ante hoc explainable methods structurally embed vibration signal processing principles, fault dynamics mechanisms, and domain expert knowledge into the model during the model design and training phase, endowing the model with inherent full-process traceable explainability. These methods are characterized by mechanism embedding and inherent transparency, with explanation generation synchronized with model decision-making and high coupling with the model architecture, and their feature learning is deeply coupled with the physical laws of faults, thus fundamentally addressing black-box decision-making. Their core approaches include three categories: signal processing operator embedding, physical mechanism constraint embedding, and inherently explainable attention mechanism embedding with explicit physical mapping.

**Figure 4:**
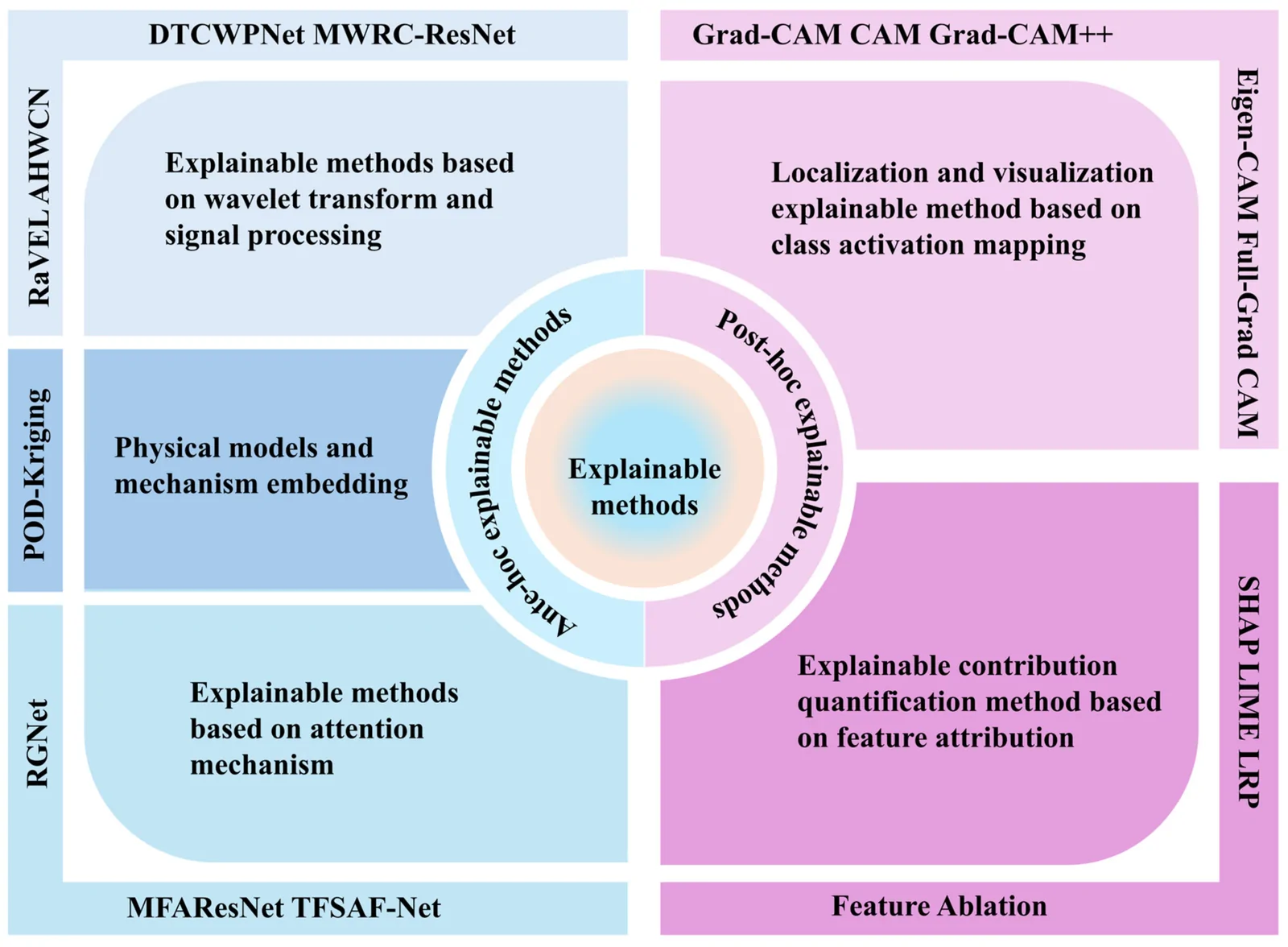
Taxonomy of explainable AI methods for rotating machinery fault diagnosis.

Post hoc explainable methods do not alter the core inference architecture and pre-trained parameters of the original black-box model. These methods feature model training process decoupling and post hoc tracing, are independent of the original model’s training process without the need for reconstruction or full retraining, and have strong universality. A small number of methods only require lightweight adaptation of the model output layer, making them suitable for mature black-box models already deployed in industrial field sites. Their core approaches include two categories: visual localization via Class Activation Mapping (CAM), and contribution degree quantification via feature attribution. This classification system aligns with the technical logic of industrial rotating machinery FD, and its technical branches and typical representative methods of each category are illustrated in Figure 4.

The principal XAI algorithms differ in their explanation mechanisms, output forms, and underlying assumptions. Ante hoc models based on signal-processing operators generate physically meaningful features, such as fault-sensitive frequency bands, wavelet coefficients, or order components, during inference. Physics-guided models further constrain feature learning or prediction using governing equations, fault mechanisms, or expert knowledge. Attention mechanisms assign data-dependent weights to channels, time steps, or frequency bands, although high attention weights do not necessarily constitute faithful explanations. Among post hoc methods, CAM and Grad-CAM localize class-relevant regions using feature activations and target-class gradients. LIME approximates individual predictions through locally fitted surrogate models, whereas SHAP estimates feature contributions according to Shapley-value principles. Integrated Gradients accumulates gradients from a reference baseline to the input, while LRP and DeepLIFT propagate relevance or activation differences backward through the network. These methods consequently produce explanations at different levels, ranging from physically meaningful signal components and attention distributions to activation maps and numerical feature contributions.

These methodological differences directly affect computational overhead and deployment suitability. Signal-processing-embedded models can generate diagnostic evidence during normal inference, but complex wavelet banks or iterative decompositions may increase model size and memory demand. Attention and CAM-based methods generally require limited additional computation, although storing intermediate activations can be costly for long vibration sequences or high-resolution time-frequency maps. Gradient-based attribution methods require backward propagation and may be sensitive to baseline selection or network structure, whereas perturbation-based methods such as LIME and sampling-based SHAP repeatedly evaluate modified inputs and are therefore less suitable for continuous microcontroller-side explanation. Method selection should consequently consider not only explanation form, but also input length, explanation frequency, memory consumption, latency, energy demand, physical consistency, and robustness to explanation drift. Table 3 summarizes these trade-offs and distinguishes methods suitable for continuous edge diagnosis from those more appropriate for offline auditing or expert analysis.

As shown in Table 3, intrinsically interpretable approaches based on signal processing or physical knowledge generally provide better physical consistency and lower explanation overhead, making them relatively suitable for resource-constrained deployment. Attention and CAM-based methods offer convenient visualization but require additional fidelity and stability validation because visually concentrated explanations are not necessarily reliable. Perturbation-based methods, particularly LIME and SHAP, provide flexible post hoc explanations but involve repeated model inference and may exhibit explanation instability, which limits their direct online implementation on microcontrollers. Therefore, method selection should jointly consider explanation quality, computational resources, and the intended deployment scenario rather than interpretability alone.

Accordingly, Table 3 serves as a method-selection guide, while the subsequent sections examine whether these methods remain reliable under industrial operating constraints and whether their outputs can ultimately support practical maintenance decisions.

### 3.3. Scenario Constraints for Industrial Deployment of Explainable Diagnostic Methods

The aforementioned two core categories of ante hoc and post hoc explainable methods form the mainstream technical framework of explainable FD for rotating machinery, and relevant methods have achieved extensive and reliable validation on public standard laboratory datasets. However, there are inherent differences between the industrial deployment scenarios of rotating machinery intelligent FD and controlled laboratory environments. The requirement for explainable diagnostic methods to adapt to the objective constraints of industrial field sites constitutes the core boundary between laboratory theoretical research and practical engineering deployment.

Existing studies have improved the physical explainability of diagnostic models by designing specialized explainable feature extraction modules. Nevertheless, such methods usually take the increase in model structural complexity as the core cost, which reduces the deployment convenience and real-time inference efficiency of the model to a certain extent [30]. For industrial scenarios, most diagnostic models need to be deployed on low-cost embedded edge devices with extremely stringent constraints on computing power, storage space, and power consumption. In contrast, most existing physical explainability enhancement methods based on module embedding have high structural complexity and excessive computational overhead, which makes it difficult to realize real-time inference and deployment on the edge side. Meanwhile, conventional model lightweighting processes often face the inherent trade-off among model lightweighting, diagnostic accuracy, and explainability: improper lightweighting operations such as unstructured random pruning may lead to the simultaneous degradation of model explainability and diagnostic accuracy, thus forming a trilemma that is difficult to balance in industrial edge deployment scenarios.

The actual operation of industrial rotating machinery is generally faced with complex working conditions including strong background noise and wide-range variable speed/load. Most existing explainable FD methods can only maintain stable diagnostic and interpretation performance under fixed operating conditions. When the operating conditions change, the data distribution of the original vibration signal shifts significantly. This makes it difficult for existing methods to meet the practical diagnostic requirements of complex industrial scenarios [31].

In view of the aforementioned core constraints of industrial scenarios, Section 4 systematically combs through the research progress of ante hoc and post hoc explainable methods in the field of rotating machinery FD, with a core focus on how existing studies respond to the above engineering constraints. On this basis, it summarizes the specialized industrial deployment-oriented optimization technologies for explainable FD, and completely presents the technical evolution roadmap of explainable methods from laboratory theoretical research to industrial engineering adaptation.

## 4. Applications of Explainable Methods in IFD of Rotating Machinery

Based on the unified two-category classification system of ante hoc and post hoc explainable methods constructed in Section 3.2, this section systematically combs through the research progress, typical applications, and engineering practices of mainstream explainable methods in rotating machinery FD. Aiming at the core engineering constraints of industrial FD scenarios summarized in the previous section, this section focuses on the industrial deployment adaptability of various methods while completing the review of technical progress.

**Figure 5:**
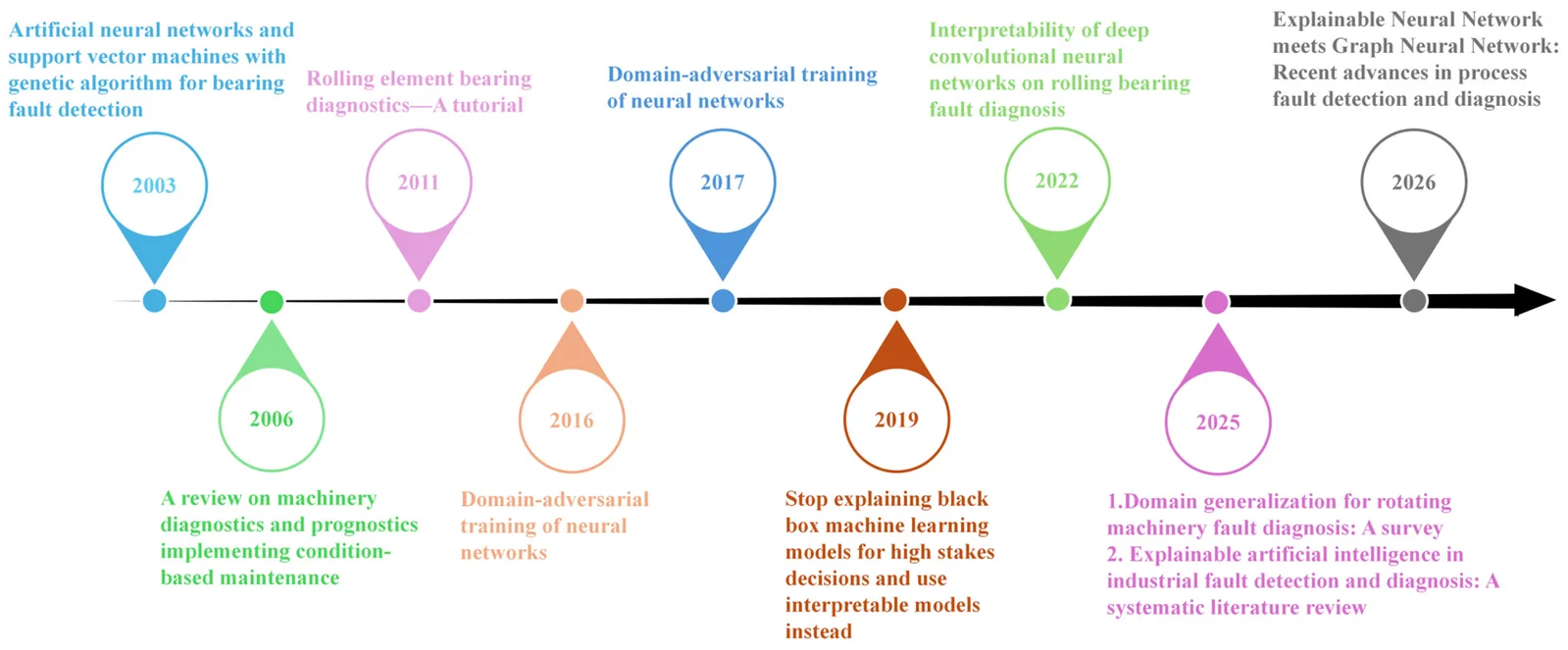
Timeline of XAI development in rotating machinery fault diagnosis.

We review the 2003-to-date progress of intelligent fault diagnosis for rotating machinery, which has undergone three sequential development stages. The initial traditional diagnosis stage established core signal processing methods and a standardized technical route of manual feature engineering combined with shallow machine learning models, laying the field’s methodological foundation [32,33,34]. The subsequent deep learning stage realized end-to-end fault diagnosis via deep models such as CNNs, and solved key generalization challenges through transfer learning and domain adaptation, promoting the engineering application of deep learning-based diagnosis [35,36]. The latest explainable diagnosis stage focuses on addressing the “black-box” defect of deep learning, with Grad-CAM, SHAP, and other XAI methods widely used to improve model transparency, while technologies including physical-data fusion, remaining life prediction, and model lightweighting have advanced to enhance industrial applicability [37,38]. Recent reviews have separately examined domain generalization in rotating machinery [39] and XAI frameworks for industrial and process fault diagnosis [40,41], collectively motivating further research on explainability, cross-condition robustness, lightweight implementation, and industrial deployment. The technological development roadmap of the three stages is illustrated in Figure 5.

### 4.1. Ante Hoc Explainable Methods

The core of ante hoc explainable methods lies in realizing the inherent transparency of the model through the embedding of mechanism priors, aligning the model’s decision-making logic with the physical fault mechanisms, and fundamentally avoiding the inherent defects of black-box models. This section systematically reviews the research progress of such methods from three dimensions: signal processing operator embedding, physical mechanism constraint embedding, and explicit mapping of attention mechanisms.

#### 4.1.1. Explainable Methods Based on Wavelet Transform and Signal Processing

Vibration-based fault diagnosis relies on signal representations that expose the frequency, time, and transient characteristics of mechanical faults. The Fast Fourier Transform (FFT) converts a time-domain signal into its global frequency spectrum and is useful for identifying stationary characteristic frequencies, harmonics, and sidebands. However, FFT does not indicate when a frequency component occurs. The Short-Time Fourier Transform (STFT) applies a sliding window to obtain a time-frequency representation, but its time-frequency resolution is restricted by the selected window length. The Discrete Wavelet Transform (DWT) provides multiresolution decomposition through a discrete set of scales, making it suitable for transient impulses and multiscale fault features. The Continuous Wavelet Transform (CWT) evaluates signal similarity over continuously varying scales and is commonly used to generate time-frequency maps for deep-learning models [42,43].

Synchrosqueezed wavelet and related reassignment transforms further concentrate dispersed time-frequency energy and improve the localization of rapidly varying components in non-stationary rotating-machinery signals [44,45]. For variable-speed machinery, order-domain analysis relates spectral components to rotational speed and can reduce the influence of speed fluctuations [46]. These representations are not merely preprocessing tools. Their outputs can be related to physical fault evidence, such as periodic impulses, bearing characteristic frequencies, gear-meshing frequencies, modulation sidebands, and transient energy concentration. Therefore, embedding FFT, STFT, DWT, CWT, or order-tracking operations into a diagnostic model can improve both feature extraction and physical traceability [47].

This section focuses on the first subcategory of ante hoc explainable methods defined in Section 3: signal processing operator embedding methods. The core of such methods is to embed signal processing operators with explicit physical meanings into deep neural networks during the model design and training phase, transforming the feature extraction process from a data-driven black-box operation into a traceable, comprehensible physical transformation with inherent explainability. The core idea of these methods is to leverage the time-frequency localization characteristics of wavelet transform, the frequency band selection mechanism of filters, and the adaptive decomposition capability of modal decomposition methods, to endow DL models with prior knowledge consistent with fault physical mechanisms.

Random wavelet kernel convolution improves physical interpretability by replacing conventional CNN kernels with trainable parameterized wavelet bases, making time-frequency feature extraction intrinsically traceable. On bearing and gearbox datasets, it outperformed CNN baselines in accuracy and inference efficiency under noise, limited samples, and class imbalance [48]. The multi-superlet CNN embeds an adaptive frequency-learnable superlet layer with multi-kernel fusion and channel attention, improving the time-frequency resolution of modulated fault signals while retaining ante hoc interpretability [49]. Extending wavelet embedding beyond a single layer, the attention-guided hierarchical wavelet convolution network combines hierarchical decomposition with Autogram-based fault-band selection and hybrid gradient-attention weighting, aligning learned features with fault characteristic frequencies during forward propagation [50].

To integrate wavelet analysis into model training rather than use it only as preprocessing, MWRC-ResNet removes DWT downsampling, applies adaptive padding, and embeds trainable wavelet modules in each ResNet layer. On the CWRU and HIT datasets, it retained accuracies of 90.27% and 96.33%, respectively, at an SNR of −15 dB, outperforming ResNet and VGG baselines [51]. A dual-tree complex wavelet packet network couples trainable wavelet convolutions with Bayesian shrinkage and energy-spectrum pooling, establishing a direct correspondence between network operations and wavelet principles. It achieved the highest accuracy among the compared methods across different SNR levels and superior signal-reconstruction quality, demonstrating robust and physically interpretable feature extraction [52]. A wavelet-prior-guided algorithm-unrolling network further integrates wavelet priors with iterative learning, whose basis functions align with fault-characteristic orders and support ante hoc interpretability [53].

To enhance weak fault-related frequency components under strong noise, a wavelet-based spectrum augmentation network embeds learnable CWT filter kernels into model training. An adaptive multi-scale weighting mechanism enhances fault-sensitive frequency bands while suppressing noise-dominated components, providing physically interpretable feature extraction based on wavelet-spectrum principles. Experiments on the XJTU-SY and aero-engine bearing datasets under different SNR levels demonstrated higher diagnostic accuracy than the compared methods. Feature visualization further showed that the learned filters aligned with bearing fault characteristic frequencies, supporting the effectiveness and physical interpretability of the proposed mechanism [54]. The above wavelet frequency domain enhancement methods achieve fault feature enhancement in conventional strong noise scenarios by directionally enhancing fault-related frequency bands and suppressing noise interference. However, in extreme strong noise environments with lower SNR, the strategy relying solely on passive noise suppression is still difficult to restore early weak fault features completely submerged by noise.

**Figure 6:**
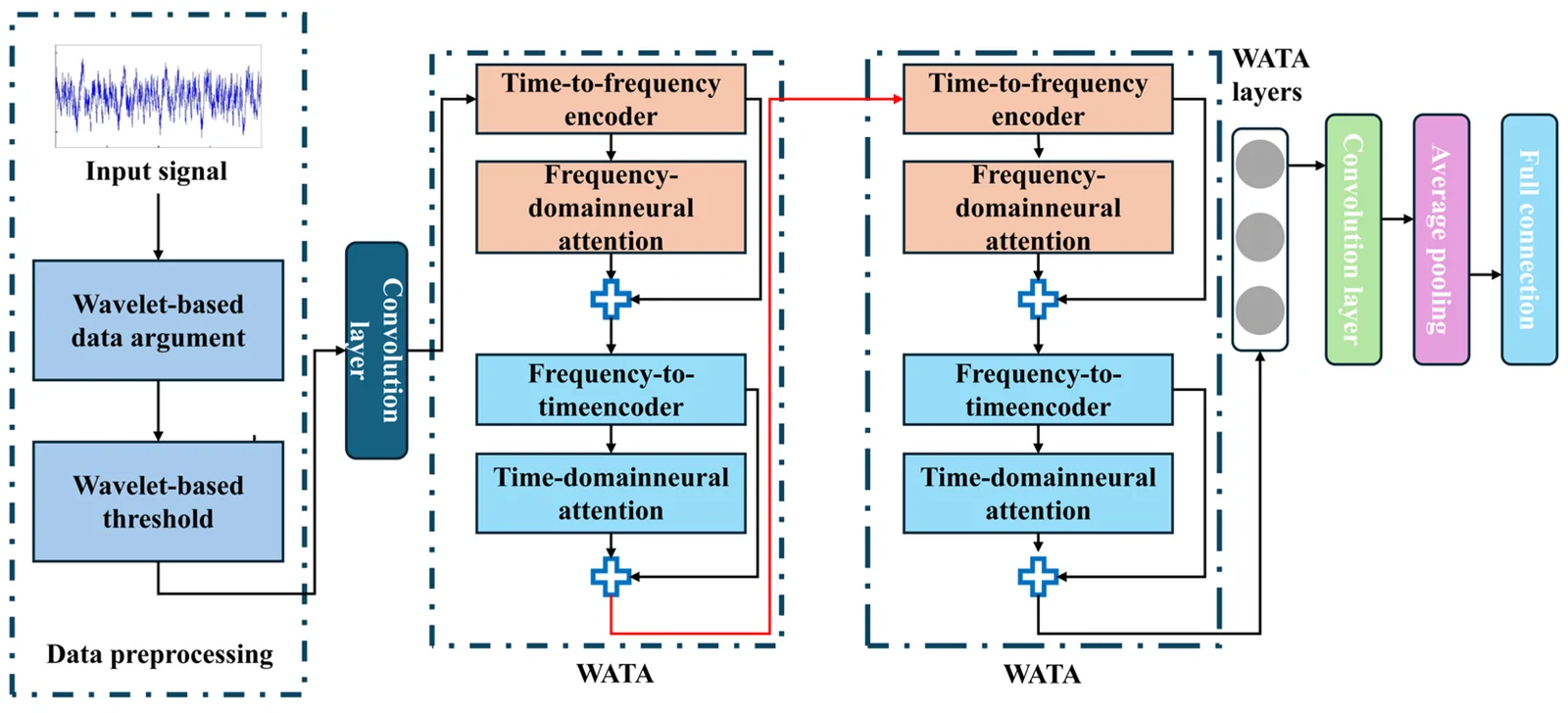
WATA-SRN network architecture diagram.

To address this limitation, relevant studies break through the traditional technical framework centered on noise elimination and propose a wavelet and time-domain attention-guided stochastic resonance network, which transforms noise from “interference to be eliminated” into “an available tool for feature enhancement” [55]. Its network framework diagram is shown in Figure 6.

A mitigating degree-bias graph neural network was developed to improve noise robustness, information aggregation, and interpretability in rotating machinery fault detection. The method applies FFT-based frequency-domain decoupling and sparsity-driven spectral selection to retain fault-sensitive components while suppressing noise. A degree-bias mitigation mechanism further improves graph information aggregation, enabling traceable feature extraction and decision-making. Experiments on aviation bearing, planetary gearbox, and real aero-engine datasets achieved 86.09% accuracy under severe noise with only 0.083 million parameters, demonstrating robustness, lightweight computation, and physical interpretability [56].

To address the problem of insufficient inherent explainability of DL models in rolling bearing FD, relevant studies integrate the core principle of Short-Time Fourier Transform (STFT) into convolutional kernel design and propose a modularized short-time time-frequency kernel network. This method realizes adaptive extraction of explainable local time-frequency features through the combination of a Gaussian window function and frequency-selective convolutional kernels, and achieves a classification accuracy of 95.91% on the Paderborn University Bearing Dataset, which significantly outperforms various comparative mainstream CNN models [57].

To improve fault-frequency-band extraction, noise robustness, and explainability, Differgram uses convex optimization to identify discriminative bands and combines logistic regression with change-point analysis for transparent band selection. Tests on public bearing datasets confirmed accurate localization of single and compound faults and stronger noise robustness than fast spectral kurtosis [58]. For non-stationary signals, Synchrosqueezed Wavelet Transform (SWT/SST) provides high time-frequency resolution with reduced frequency mixing. Its integration with deep learning has enabled explainable diagnosis of hydraulic pumps and rolling bearings [59,60].

Recent signal-to-image approaches have extended explainable feature extraction to early weak-fault diagnosis. Wang et al. developed an optimized recurrence plot that combines asymmetric multiscale reconstruction, an information-based threshold, and directional encoding to preserve the temporal evolution of weak bearing-fault features. When integrated with an improved residual network, the method achieved a reported improvement of 5.61% and used Grad-CAM to identify the recurrence structures supporting the diagnostic decision [61]. Compared with wavelet-based methods, recurrence plots provide an intuitive representation of state-space evolution. However, their results remain sensitive to embedding and threshold parameters, while Grad-CAM provides post hoc rather than inherently physical explanations.

Overall, signal-processing-embedded models offer a stronger physical correspondence than generic CNNs because their filters, frequency bands, and decomposition paths can be related to fault characteristic frequencies. They are particularly suitable when the fault mechanism is reflected by stable periodic impulses or identifiable time-frequency components. Their robustness to additive noise is generally higher than that of unconstrained data-driven models, but this advantage assumes that fault information remains within the selected basis or frequency range. Changes in speed, transmission path, sensor bandwidth, or installation may shift the relevant components and reduce cross-machine generalization. Computational cost also varies substantially: compact learnable filters and operator networks are compatible with edge inference, whereas dense time-frequency maps, multi-scale wavelet banks, and iterative decomposition increase memory and latency. Thus, physical alignment should be verified under operating-condition and sensor-domain shifts rather than inferred from visualization on a single laboratory dataset.

#### 4.1.2. Explainable Methods Based on Physical Models and Mechanism Embedding

Explainable methods based on physical models and mechanism embedding aim to directly incorporate the physical mechanisms of rotating machinery—such as rotor dynamics, fault characteristic frequencies, and signal modulation principles—in the form of equations, constraints, or parameterized structures, into the design and training process of deep networks. This ensures that the model’s decision-making aligns with physical laws, thereby achieving inherently explainable diagnostics consistent with the ante hoc classification criteria defined in Section 3.2.

Conventional data-driven models capture input–output correlations without explaining system dynamics. To improve the physical interpretability of axial piston pump diagnosis, a PINN-based framework embeds the governing pipeline-fluid equation into the loss function and uses measured pressure pulsations to virtually estimate outlet flow pulsation. Residuals between predicted and measured signals identify slipper and cylinder-block faults, reduce dependence on labeled data, and provide mechanistic evidence for diagnostic decisions [62].

Under time-varying speeds, a prior-knowledge-guided mode filtering network encodes rotational speed into polynomial-fitted FIR kernels for adaptive order tracking and speed-sensitive mode extraction. A pooling layer incorporating twelve time-domain indicators further aligns feature statistics with established FD practice, achieving over 97% average accuracy on gearbox and UO bearing datasets [63]. For aircraft engines under noisy and non-stationary conditions, a refined time-frequency network transforms CNN kernels into interpretable time-frequency kernels using a higher-order phase operator, enabling adaptive feature extraction and noise suppression [64].

To reduce the computational burden of physical models, a machine-learning-based real-time CFD simulation method was proposed for axial piston pump condition monitoring [65]. A high-fidelity 3D transient CFD model was used to generate healthy outlet pressure pulsation data, and a POD-Kriging surrogate model was built to predict signals efficiently. With the coefficient of determination between measured and predicted signals as the health indicator, simulation time was reduced from 7.2 h to 1.2 s while enabling fault detection. For rotor fault parameter quantification, a surrogate framework with epistemic uncertainty quantification was proposed [66]. Based on a Bayesian deep operator network, Fourier feature expansion and POD were introduced as physical priors to improve explainability and mitigate spectral bias. A two-step optimization strategy combining Bayesian optimization and gradient descent addressed non-convexity and non-uniqueness, yielding average inversion errors of 3.01% and 3.89% for angular and parallel misalignment, respectively, while identifying multiple feasible parameter sets.

**Figure 7:**
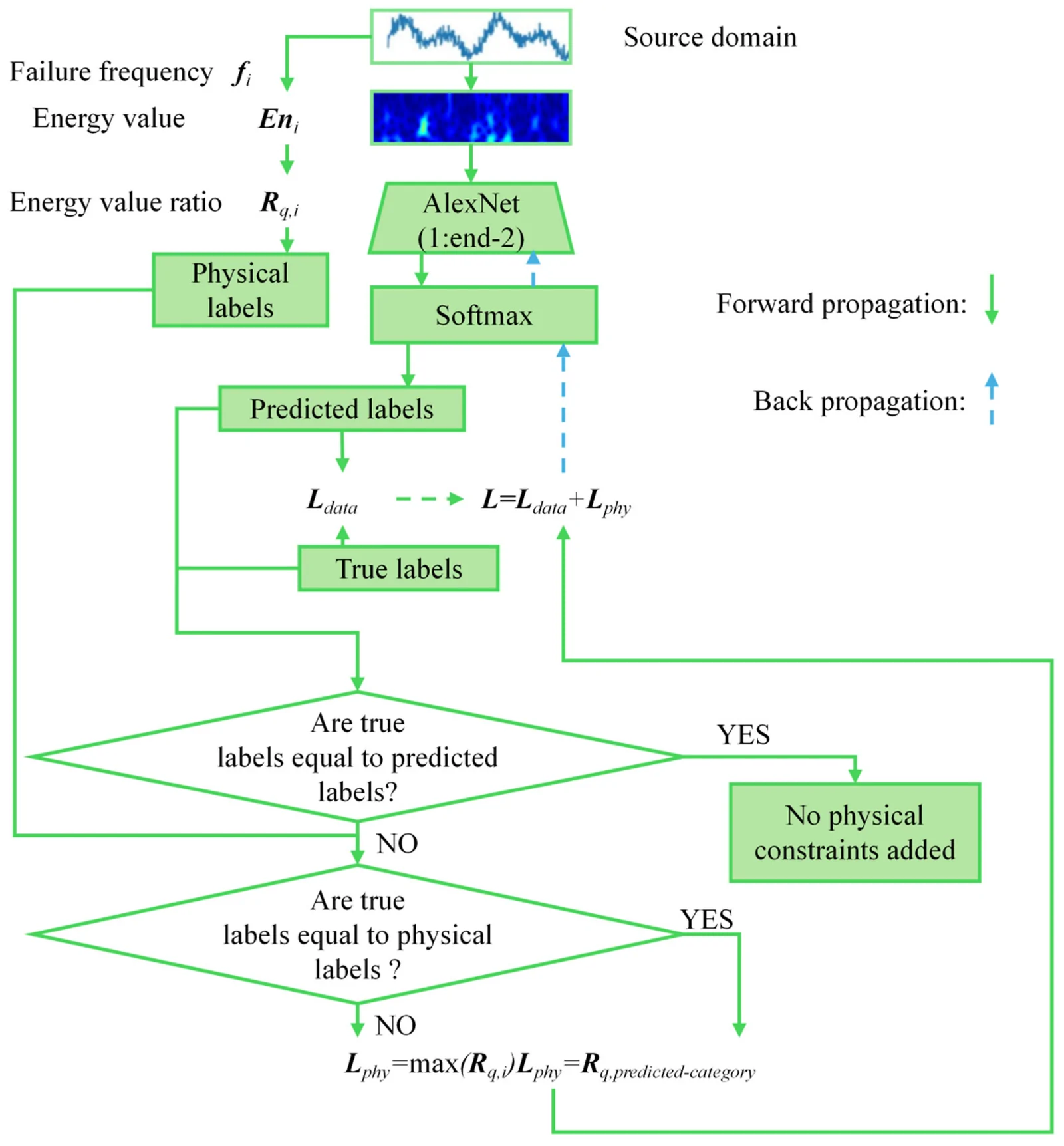
The architecture of the source domain model [67].

For the industry pain points in cross-machine bearing fault diagnosis, including insufficient labels in the target domain, difficult knowledge transfer, and weak explainability, a physics-informed unsupervised domain adaptation framework is proposed. In the target domain, it refines pseudo-labels through a dynamic correction mechanism to improve reliability. The architecture of the source domain model is clearly illustrated in Figure 7. Results show that physics-informed unsupervised domain adaptation achieves higher diagnostic accuracy and F1 score than traditional domain adaptation methods, demonstrating its effectiveness in explainable cross-machine fault diagnosis [67].

For imbalanced few- and zero-shot FD, a physics-prior-guided generation framework combines a five-degree-of-freedom nonlinear rotor-bearing model with Hertzian contact theory to create physically consistent fault samples. A threshold Wasserstein GAN narrows the simulation-to-real distribution gap by incorporating healthy target-machine signals. In zero-shot transfer from 800 to 1000 RPM, a CNN trained on the generated samples achieved 97.35% accuracy, 46.35 percentage points higher than training on raw simulated data, supporting mechanism-aligned feature learning and ante hoc explainability [68].

For small-sample axial piston pump diagnosis, a UG/Simcenter 3D-AMESim co-simulation model generates mechanism-informed data for a meta-transfer architecture integrating DNNs, ResNet, channel-spatial attention, and domain adaptation. The method achieved 98.1% average accuracy with ten labeled samples per class and retained 97.5% accuracy under strong noise [69].

A transparent operator network replaces black-box neurons with interpretable signal-processing operators. Its learnable Morlet wavelet filters have physically meaningful center frequencies and bandwidths, while gating, skip connections, and statistical features such as negentropy, kurtosis, and mean value provide traceability from vibration signals to linear classification. With only 208 trainable parameters, the model achieved 100% accuracy on a constant-speed bearing dataset and 55.2% in a variable-condition generalization task trained at 10 Hz and tested at 1 Hz, while retaining strong noise robustness [70].

Focusing on mechanism-embedded explainable FD, another study introduced a neuro-symbolic deep expert network [71]. This method encodes domain mechanism knowledge into interpretable symbolic operators and embeds them into the network at the design stage. Through sparsity learning, it generates a fully white-box diagnostic decision path guided by mechanism priors. The model achieves over 97% average accuracy on public bearing and gearbox datasets and maintains stable performance under strong noise conditions with SNR as low as −10 dB, demonstrating both high diagnostic accuracy and strong robustness.

For coupled bearing–gearbox FD, a physics-inspired deep learning network based on dilated Kronecker convolution was proposed to address weak bearing-feature masking by strong gearbox impulses, nonlinear coupling effects, and limited explainability of pure data-driven models [72]. The method embeds physically meaningful modules, including DWT and Volterra series expansion, into the network architecture. The Volterra series characterizes the nonlinear input–output relationship of the bearing–gearbox coupled system, enabling interpretable decoupling of coupled fault features. Fault classification is then performed by combining a Kronecker convolutional feature pyramid with a bidirectional LSTM. With end-to-end inherent explainability, the method achieves 99.96% accuracy in coupled bearing–gearbox fault classification, outperforming comparable models in both diagnostic performance and lightweight design.

Compared with signal-processing embedding, physics- and mechanism-guided models provide stronger constraints on whether learned relationships are physically plausible and can reduce dependence on labeled fault samples. They are most appropriate for safety-critical systems whose governing equations, fault frequencies, boundary conditions, or component relationships are sufficiently known. Their principal limitation is model mismatch: inaccurate parameters, simplified dynamics, or unmodeled coupling can impose a biased prior and suppress genuinely informative data patterns. PINN and high-fidelity simulation approaches may also require costly optimization or offline computation, although surrogate models and operator networks can substantially reduce inference time. Consequently, these methods may generalize well across conditions governed by the same mechanism, but their transfer across machine structures or unknown fault modes is not automatic and should be supported by uncertainty analysis and model-discrepancy tests.

#### 4.1.3. Explainable Methods Based on an Attention Mechanism

The explainable network based on an attention mechanism aims to endow the model with the ability to select key information and explicitly present the decision basis in the form of attention weights, so as to convert the “focus of attention” of the model into human-understandable explanations. The core idea of this type of method is that the attention distribution learned by the model during the training process should be consistent with the physical nature of the fault.

Channel attention weights convolutional feature channels to emphasize frequency- or time-domain patterns relevant to fault mechanisms. The time-frequency spectral feature enhancement and attention fusion network (TFSAF-Net) combines nonlinear enhancement of wavelet maps, physics-guided feature-noise decoupling, and multiscale attention fusion. Its attention weights map to fault-sensitive frequency bands, while experiments on the PU dataset reported accuracies of 99.254% without noise and 90.62% at −6 dB SNR [73]. A residual global context shrinkage network integrates dispersion-entropy-based multi-sensor fusion, global-context attention, and adaptive soft thresholds to capture long-range dependencies and suppress channel-wise noise. It achieved accuracies of 98.81% without noise and 86.11% at −6 dB on self-collected bearing and gearbox datasets, with layer-wise visualization tracing feature extraction and noise suppression [74].

For small-sample diagnosis under variable conditions, MFAResNet fuses multidirectional vibration signals through principal component analysis and extracts 28-dimensional time-frequency features using an attention-guided residual architecture. It achieved 98.80% diagnostic accuracy and 98.32% cross-condition transfer accuracy, while its attention weights provided feature-level interpretation [75]. For hydraulic pumps, multi-head attention integrated with ResNet and transfer learning focuses on weak fault impulses in long sequences, with attention maps exposing the fault-sensitive features supporting model decisions [76].

For IoT-based rotating machinery PHM, an explainable parallel spatial CNN-LSTM processes raw time-domain signals and CWT maps through dual branches, combines them using a physical-information fusion layer, and applies self-attention to fault-periodic time windows. It achieved 100% accuracy on a self-collected gearbox dataset, retained over 90% accuracy with only 10% of the training data, and reached 100% in multi-condition tests on the CWRU dataset [77]. For unsupervised cross-domain bearing diagnosis, an explainable subdomain-enhanced adaptive network integrates sparse-subsegment denoising, lightweight multi-feature extraction, and improved local maximum mean discrepancy. It achieved over 98.3% average accuracy with only 30 training samples on the CWRU and LUT datasets while maintaining noise robustness [78]. A compressed-attention encoder-transformer-decoder further uses dynamic attention to fuse acoustic and vibration signals and expose fault-sensitive features, achieving 99.31% accuracy on a self-collected bearing dataset and 91.26% at −6 dB SNR [79].

CNN-Transformer architectures can jointly capture local fault impulses and global temporal dependencies. A lightweight double-prioritized-experience-replay agent combines a CNN front end with a multi-head-attention Transformer Q-network for local feature extraction and long-range modeling. It outperformed ResNet, DenseNet, and standard Transformers in small-sample tests on 6203 and electric-locomotive bearing datasets, while kernel-density visualization of Q-values revealed class-specific decision confidence [80].

For RUL prediction, a dual-region-guided multi-head attention mechanism uses an auxiliary constraint to separate local heads, which capture abrupt degradation changes, from global heads, which model long-term trends. Combined with channel attention and a dual-channel temporal convolutional encoder–decoder, its attention maps and t-SNE visualizations provide insight into the degradation-learning process [81]. For electrical-erosion diagnosis of traction-motor bearings, a bidirectional simple recurrent unit-Transformer jointly models local and global temporal features, achieving 99.8% overall accuracy, over 98% for each fault class, and over 96% under cross-load conditions. Layer-wise t-SNE visualization further traces the progression from mixed inputs to discriminative fault features [82].

A dual-channel fuzzy CNN integrates fuzzy logic with deep learning for gearbox diagnosis. Its fuzzy channel uses Gaussian membership functions and rule layers to model uncertainty, while the CNN channel extracts local and global features from type-II-fuzzy-enhanced time-frequency maps. Layer-wise relevance propagation quantifies neuronal contributions to each decision. The model achieved 98.06% average accuracy and retained 96.22% at −2 dB SNR, while relevance heatmaps aligned the learned evidence with gear-fault vibration mechanisms [83].

A dense fusion attention network addresses noise-induced feature masking and weight interference in cascaded attention by embedding dispersed attention within dense connections. Zoomed spatial self-attention and CAM independently calibrate spatial and channel features, while attention-dynamics and t-SNE visualizations relate the feature-learning process to diagnostic outcomes [84].

Recent studies have extended attention-based interpretation toward multi-source industrial monitoring. Xie et al. developed a co-evolutionary decoupled attention framework for weak thermal fault diagnosis of marine diesel engines. The method combines physically informed trend–mean feature decoupling, channel–spatial attention, and Fusion-CAM, with occlusion experiments used to evaluate whether the highlighted thermal variables contribute to the decision [85]. Wan et al. integrated VMD, a one-dimensional CNN, and dual channel–spatial attention for aero-engine bearing diagnosis under varying loads and noise. The framework achieved an accuracy above 98.5% and was implemented in a real-time client–server monitoring system with confidence-based rejection of ambiguous samples [86]. These studies show that attention can support feature prioritization and human inspection; nevertheless, attention weights alone should not be regarded as causal explanations unless their fidelity is independently validated.

In comparison with physics-embedded models, attention mechanisms are easier to integrate into CNNs, recurrent networks, Transformers, and multi-sensor fusion architectures, and channel or temporal attention usually adds only limited inference overhead. They are therefore suitable for feature selection, sensor weighting, and localization of informative time-frequency regions. However, their explainability rests on the assumption that attention weights faithfully represent the evidence used for prediction; this assumption is not guaranteed, because different attention distributions may produce similar outputs and may drift under noise or domain shift. Multi-head and Transformer attention further increase memory and computational requirements. Attention-based models can generalize across architectures, but the learned weights are often dataset-dependent. Their explanations should therefore be validated through ablation, perturbation, occlusion, or agreement with fault-characteristic frequencies before being used as engineering evidence.

### 4.2. Post Hoc Explainable Methods

In the engineering implementation of rotating machinery FD, post hoc explainable methods are indeed more readily accepted by field practitioners. Most of the intelligent diagnostic systems that have been large-scale deployed in current industrial sites are trained black-box models based on mature architectures such as CNN and Transformer, whose diagnostic accuracy and robustness under extreme conditions such as strong noise and variable working conditions have been fully verified. Reconstructing these models with ante hoc methods to introduce explainability not only requires significant cost investment in model retraining, field debugging, and compliance re-certification, but also is likely to sacrifice part of the original diagnostic performance while improving explainability. In contrast, post hoc explainable methods can directly interface with in-service black-box models and restore the decision-making logic without any modification to the model itself. This not only retains the performance advantages of the original model, but also meets the requirements of traceable, verifiable, and credible diagnostic conclusions in industrial safety-critical scenarios, so it is most widely used in engineering applications.

In terms of the output form and functional positioning of post hoc explainable methods, current research in the field of rotating machinery FD can be roughly divided into two directions. One is the visualization method based on CAM. The core of this type of method is to map the model’s decision basis in the high-dimensional feature space back to the original input space through CAM technology, and finally intuitively present the time-domain segments, frequency band intervals, or key time-frequency regions that the model focuses on during diagnosis in the form of heatmaps or saliency curves. This makes it possible to clearly verify whether the model’s focus corresponds to the physical characteristics of the fault, thus providing a visual basis for decision-making. The other is the quantitative method based on feature attribution. This type of method focuses on using mathematical tools such as gradient, perturbation, or game theory to quantitatively analyze the contribution weights of input features, hidden layer neurons, and even different sensor channels to the final diagnostic conclusion. Through this analysis, it can be clarified which features have a positive supporting effect on the conclusion, which cause interference, and which are completely irrelevant, thus providing a quantitative causal explanation for the model’s decision-making logic. These two types of methods, one focusing on qualitative visualization and the other on quantitative attribution, complement each other from different perspectives and together form a complete technical system for post hoc explainability.

#### 4.2.1. Localization and Visualization Explainable Method Based on CAM

The localization and visualization technology based on CAM and its derivative methods is the most representative branch of post hoc explainable methods. Its core principle is to generate a class activation heatmap with the same dimension as the input signal by performing global average pooling on the feature map output from the last convolutional layer of the DL model, combined with the classification weights of the fully connected layer. The weight value of the heatmap directly reflects the contribution of different regions in the input signal to the model’s final classification decision, thus realizing accurate localization of the key regions for the model’s decision-making. In the rotating machinery FD scenario, this type of method can directly correlate the model’s decision basis with physical features such as periodic impulses and characteristic frequencies caused by faults in the vibration signal, breaking the “black box” property of deep learning models. It can verify whether the model has learned effective features matching the fault mechanism, rather than spurious correlation information such as noise and interference, thus becoming the mainstream method for explainability analysis of FD models.

Early studies mainly focused on introducing CAM and its basic improved variant, Grad-CAM, into the field of rotating machinery FD, and established a basic analysis framework from CAM visualization to fault physical feature verification. Some studies converted vibration signals into time-frequency maps via STFT, fed them into a CNN model for fault classification, and generated activation heatmaps corresponding to various fault types through CAM. The results showed that when the model identified inner race faults and outer race faults, the frequency bands it focused on were highly consistent with the theoretically calculated ball pass frequency of inner race and ball pass frequency of outer race, respectively. This verified the consistency between the features learned by the model and the physical fault mechanism, and provided an intuitive basis for improving the explainability of DL diagnostic models [87]. A post hoc explainability analysis method based on Generative Adversarial Networks (GANs) is proposed. Specifically, an eigenvalue decomposition-based Eigen-CAM module is embedded into the classifier. Singular value decomposition is conducted on the convolutional feature maps to generate class activation heatmaps corresponding to the time-domain vibration signals, which can intuitively visualize the signal regions focused by the model during fault classification. The results show that the high-attention regions of the model are in good agreement with the theoretical fault period calculated from the bearing geometric parameters and rotational speed. This study realizes the post hoc explanation of the decision-making basis of the DL model from the perspective of visualization, and provides methodological support for enhancing the credibility of FD models [88]. To further expand the analytical dimension of explainability, existing studies have combined Grad-CAM visualization with frequency-domain feature analysis. These studies locate the sensitive regions that the model relies on for decision-making via CAM, and conduct comparative verification between these regions and the fault characteristic frequencies identified through frequency-domain analysis. The results demonstrate that the spatial regions focused on by the model are highly consistent with the data channels containing the dominant fault frequencies. This approach achieves bidirectional verification between time-domain localization and frequency-domain mechanism explanation, and enriches the explainability connotation of CAM methods [89].

CAM-based analysis has expanded from conventional CNN visualization to GANs, autoencoders, quadratic networks, and lightweight diagnostic architectures. For shaft-orbit images, a VAE-GAN-based blade-fouling detector incorporates invariant-moment features and Grad-CAM to localize abnormal regions and trace the spatial evolution supporting anomaly decisions [90]. For quadratic neural networks, hook-based activation capture visualizes kernel responses and quadratic attention maps, while LSTM hidden-state visualization extends interpretation across the hybrid architecture [91]. A fully explainable CNN further combines interpretable signal-processing and feature-extraction layers with Grad-CAM, showing that its high-attention regions coincide with periodic impacts associated with bearing faults [92]. Similarly, Grad-CAM applied to a lightweight channel-sharing spatial-unidirectional convolutional network localized characteristic rows of rolling-element impacts, validating the physical feature-capturing ability of both its preprocessing method and convolutional layer [93].

**Figure 8:**
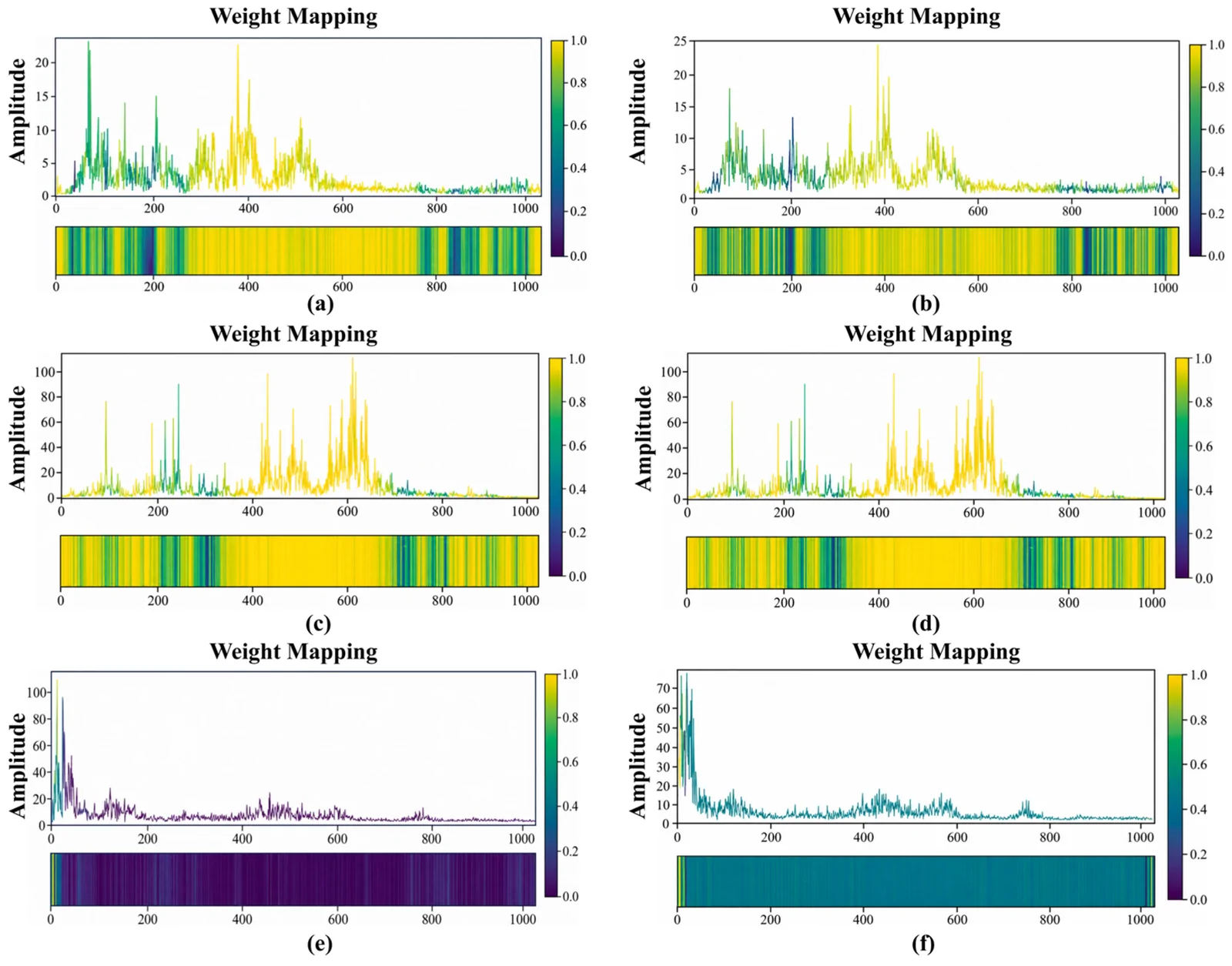
Visualization of CAM. (**a**) CWRU-RF, (**b**) CWRU-OF, (**c**) LUT-RB, (**d**) LUT-OI, (**e**) RG-CT, (**f**) RG-HT.

On the basis of research on fundamental methods and fusion optimization, relevant studies have further constructed a multi-dimensional explainability analysis system based on CAM, and extended it to more rotating machinery components such as planetary gearboxes and planetary roller screw mechanism, as well as real industrial scenarios including variable working conditions, small sample sizes, and strong noise, thus promoting the engineering application of such methods [94]. Aiming at the insufficient explainability of cross-domain FD models, a study proposed an explainable frequency-enhanced domain adaptive network by introducing Morlet wavelets for weight initialization in the initial layer of the feature extractor for incorporating physical prior knowledge. CAM is adopted to perform visualization analysis of the weight distribution of the Morlet convolutional layer, and provides explainability support for cross-domain FD models under variable working conditions [95]. The results are shown in Figure 8.

CAM-based methods are attractive because they leave the trained classifier unchanged, normally require only one forward–backward pass, and provide intuitive localization for CNN-based time-frequency images or transformed vibration representations. They are therefore useful for retrospective model auditing and for checking whether highlighted regions overlap fault periods or characteristic frequencies. However, CAM assumes that the spatial organization and final convolutional feature maps retain diagnostically meaningful localization. Its resolution is architecture-dependent, and interpolation can produce visually plausible but non-faithful heatmaps. Saliency regions may also change under noise, rescaling, or small input perturbations even when the predicted class is unchanged. Consequently, CAM has low-to-moderate computational cost and broad applicability to convolutional models, but limited quantitative comparability and uncertain cross-condition robustness. It should be treated as localization evidence rather than proof of causal fault reasoning, particularly for early weak faults and safety-critical maintenance decisions.

#### 4.2.2. Explainable Contribution Quantification Method Based on Feature Attribution

The core of post hoc explainable methods based on feature attribution is to quantitatively calculate the contribution and importance of different dimensions of input signals, various extracted features, different sensor channels, and even neurons in the intermediate layers of the model to the final diagnosis result via a rigorous mathematical framework, thereby enabling quantitative decomposition and attribution analysis of the model’s decision-making logic. Compared with the qualitative visualization and localization of CAM, feature attribution methods can clarify the positive promoting and negative inhibitory effects of various influencing factors on decision-making in numerical form. These methods can not only verify whether the model has learned core features consistent with the fault mechanism, but also realize automatic screening of key fault-sensitive features as well as identification and optimization of redundant model structures. Meanwhile, they provide quantitative support for fault root cause analysis, serving as a key bridge connecting data-driven diagnosis and industrial O&M decision-making.

At present, feature attribution methods in the field of rotating machinery FD are mainly divided into two technical routes. One is global and local feature attribution methods based on the game theory framework, represented by SHAP. This type of method can calculate the marginal contribution of each feature based on cooperative game theory, which has both a solid mathematical theoretical foundation and global consistency, and is the most widely used quantitative attribution method at present. The other is feature importance quantification methods based on indicators such as correlation analysis, weight statistics, and information entropy. Through statistical learning and signal analysis approaches, this type of method quantifies the contribution degree of different features, sensors, and signal components to diagnosis results, and is adaptable to more lightweight models and industrial real-time diagnosis scenarios.

SHAP-based studies have extended feature attribution across diverse industrial scenarios. In XGBoost-based bearing diagnosis, SHAP provided global feature rankings, feature-value–contribution relationships, and sample-level decision paths, identifying spectral entropy, root mean square, and impulse factor as the dominant features. Runtime tests further indicated compatibility with industrial monitoring frequencies of 200–500 Hz [96]. When integrated with graph-based diagnosis, SHAP compared time-domain, frequency-domain, and graph-structural contributions across loads and machinery types. Time-domain features dominated under low loads and in bearing diagnosis, whereas frequency-domain features were more influential under high loads and in gearbox diagnosis, enabling traceable cross-scenario attribution [97]. For marine rotating machinery under strong noise and nonlinearity, a framework combining SHAP, Grad-CAM, and spline-coefficient correlation quantified fault-specific feature contributions and related them to physical fault mechanisms [98].

In the field of feature contribution quantification based on statistical learning and signal analysis, relevant studies have constructed a multi-dimensional, full-scenario quantitative attribution system focusing on practical industrial requirements such as multi-source information fusion, transfer learning, lightweight deployment, and unsupervised diagnosis.

For multi-source motor diagnosis, Pearson and cross-correlation coefficients were used to dynamically weight current and vibration signals. Vibration contributed up to 80.5% for mechanical faults, whereas current contributed up to 85.3% for electrical faults; their complementary weights in compound faults were consistent with physical fault evolution [99]. For label-free transfer anomaly detection, order-feature importance vectors enabled adjustment of the precision-recall trade-off, while the selected orders agreed with gearbox geometry and fault characteristics [100]. A lightweight Kolmogorov–Arnold network further used recursive attribution to rank sensor features across fault detection, classification, and severity assessment, mapping their contributions to symbolic expressions [101].

In healthy-data-only unsupervised diagnosis, correlations between learned health indicators and engineering features revealed strong associations with root mean square and fault-frequency harmonics, reaching 0.98 and supporting interpretation across fault types and degradation stages [102,103]. Another study evaluated 169 handcrafted features using seven ranking methods, including ReliefF and random forests, and identified harmonic ratio and spectral flatness as transferable features for bearing and gearbox diagnosis [104]. For cross-domain diagnosis, linear cosine attention exposed the dependence of target predictions on same-class source samples [105]. In autoencoders, sparse-coding variance regularization and principal component analysis quantified the contribution of latent components to feature learning and classification [106].

Feature-attribution methods provide different mechanisms for quantifying the contribution of input features to diagnostic decisions. LIME constructs a local surrogate model from perturbed samples, whereas SHAP estimates feature contributions using a game-theoretic framework; however, both may require repeated model inference and incur substantial computational costs for high-dimensional vibration signals [107]. Integrated Gradients accumulate gradients from a reference baseline to the input, while LRP and DeepLIFT propagate relevance or activation differences backward through the network. These methods are generally more computationally efficient than perturbation-based approaches, but their explanations remain sensitive to baseline selection, propagation rules, and model architecture [108]. Consequently, explanation quality should be evaluated quantitatively in terms of fidelity, stability, robustness, localization, and physical consistency rather than relying solely on visual inspection [109].

Table 4 summarizes quantitative metrics for evaluating whether an explanation is faithful, repeatable, physically meaningful, and comprehensible in rotating machinery fault diagnosis.

Operationally, fidelity can be measured by the change in class score after top-k deletion or insertion; robustness and stability by cosine similarity or rank correlation between attribution vectors; monotonicity by the proportion of successive confidence decreases after ranked feature removal; and localization or physical consistency by the attribution mass falling within known fault-frequency bands or fault-impact regions.

These metrics should be interpreted jointly. Fidelity may be affected by unrealistic feature masking, while robustness and stability depend on whether the applied perturbations preserve the true fault state. Localization and physical consistency require reliable fault-mechanism knowledge, and physical agreement alone does not demonstrate either model fidelity or causality.

When SHAP and Integrated Gradients produce conflicting explanations for the same vibration signal, visual plausibility alone cannot determine which explanation is more reliable. The comparison should first use the same target class, input representation, attribution resolution, and normalization procedure. Moreover, the SHAP background distribution and the Integrated Gradients baseline should represent the same physically meaningful reference state, such as healthy operation under the corresponding speed and load. Both explanations should then be evaluated using identical top-k deletion and insertion tests, repeated under noise, operating-condition variations, neighboring samples, and different model seeds. Their attributed regions should also be compared with independently calculated fault-characteristic frequencies, harmonics, sidebands, or fault-impact periods. An explanation should be considered credible only when it demonstrates adequate predictive fidelity, perturbation robustness, sample-level stability, and physical consistency. If neither explanation satisfies these criteria, the result should be referred for expert review rather than used to trigger an automatic maintenance action.

Taken together, ante hoc and post hoc approaches address different engineering requirements rather than forming interchangeable alternatives. Ante hoc methods constrain feature extraction or decision rules during model construction and are preferable when physical traceability, stable explanations, and regulatory justification are primary requirements; their disadvantages are dependence on valid prior knowledge and reduced flexibility for unknown mechanisms. Post hoc methods preserve the accuracy and certification status of existing black-box models and are more practical for retrospective auditing, but their explanations approximate model behavior and may be unstable or non-causal. A hybrid strategy is therefore most appropriate for many industrial applications: mechanism- or signal-processing-guided architectures can provide an interpretable diagnostic basis, while CAM or feature attribution can independently audit whether the trained model continues to use physically credible evidence under changing conditions. Table 3 summarizes these trade-offs in terms of computational cost, explanation drift, microcontroller suitability, and principal limitations.

### 4.3. Industrial Implementation-Oriented Explainable Diagnosis Method

The two major methods, namely ante hoc explainable and post hoc explainable methods, constitute the fundamental theoretical system for the explainability of intelligent FD of rotating machinery. However, in industrial implementation scenarios, diagnostic models are still faced with multiple engineering constraints, including limited computing power and storage capacity of edge devices, insufficient cross-device generalization ability, and the requirement of expansion to full-life cycle health management. A single ante hoc or post hoc explainable method can hardly balance multiple requirements such as accuracy, explainability, robustness, and real-time performance simultaneously. Researchers have developed industrial implementation-oriented explainability methods focusing on the pain points of industrial scenarios, promoting the transition of explainable intelligent diagnosis from theoretical research to industrial implementation.

#### 4.3.1. Lightweight Explainable Diagnosis Method Under Edge Computing Power Constraints

Industrial edge diagnostic nodes are generally subject to strict constraints on computing power, storage, and power consumption. Traditional explainable models are difficult to be directly implemented and deployed, while model lightweight design is accompanied by the degradation of feature extraction capability and the loss of explainability, forming a triple trade-off dilemma of lightweight, accuracy, and explainability. Existing studies have constructed a lightweight explainable diagnosis system adapted to edge devices centering on the core ideas of structure lightweighting, feature physicalization, and efficient inference. While compressing the model scale, it simultaneously guarantees diagnostic accuracy and decision-making explainability.

Post hoc explanation methods differ substantially in their computational requirements. Perturbation-based methods, such as LIME and sampling-based SHAP, repeatedly evaluate the diagnostic model using modified inputs. Their computational cost therefore increases rapidly with the number of perturbations and the dimensionality of the vibration signal, limiting their suitability for continuous edge-side explanation. Integrated Gradients avoids random perturbation but requires multiple gradient evaluations along the path from a reference baseline to the input, and its runtime depends on the number of integration steps. By comparison, CAM, Grad-CAM, LRP, and DeepLIFT generally obtain explanations through a forward pass followed by gradient or relevance propagation, making them more computationally efficient. However, these methods still require intermediate activation storage and depend on compatible network architectures or propagation rules. DeepLIFT should therefore be distinguished from repeatedly sampled methods: its primary deployment constraints are memory demand and architecture dependence rather than a large number of model evaluations.

To address the pain points of deploying one-dimensional CNNs on the edge, the lightweight explainable convolutional neural network with an extremely simple architecture eliminates bias terms, non-linear activation functions, and redundant pooling layers, retaining only a single-layer linear convolution and a squared global average pooling structure. The features it learns are highly consistent with the Hilbert envelope spectrum, providing clear physical explainability. The model contains only 310 parameters and achieves near 100% classification accuracy on bearing datasets, perfectly adapting to edge-side storage and computational constraints [110]. Inspired by decision trees, a purely analog circuit FD method directly maps the binary classification logic onto a programmable filter array hardware system, requiring no analog-to-digital conversion or digital computation. This method achieves 78 ms-level detection and an ultra-low power consumption of 2.1 W in motor bearing FD, while possessing complete decision-making explainability, providing a new path for ultra-low power diagnosis on the edge [111]. The quantized subtraction-convolution network accurately matches the fault characteristic frequencies of gears through a learnable sparse impulse kernel subtraction layer, achieving edge deployment with only 54 KB of memory usage and an end-to-end latency of 0.748 s, meeting the requirements of real-time diagnosis and remaining useful life prediction in industrial field sites [112]. The lightweight temporal convolutional network-CNN-efficient multi-head self-attention-mobile vision transformer parallel dual-branch network, through the embedding of physical priors and structural optimization, reduces model parameters and computational cost while maintaining high accuracy and explainability [113].

A lightweight model architecture does not necessarily indicate practical deployability because parameter count alone cannot represent memory consumption, inference latency, or energy consumption on embedded hardware. To distinguish algorithm-level lightweight design from actual edge implementation, Table 5 summarizes the deployment and industrial-validation evidence reported in representative studies. Unreported indicators were marked as “NR” rather than estimated from model architecture.

Parameter count was not converted into memory consumption because actual memory requirements also depend on numerical precision, intermediate activations, software libraries, and hardware architecture. Noise robustness based on artificially added noise does not constitute validation against electromagnetic interference or explanation drift.

Explainability should be treated as part of the end-to-end diagnostic timing budget rather than as an unrestricted offline operation. The total response time can be expressed as T_total_ = T_pre_ + T_inf_ + T_xai_ + T_comm_, where preprocessing, model inference, explanation generation, and communication must jointly satisfy the application deadline Tdeadline. The relevant deadline is determined by the diagnostic or protection loop rather than by the raw sensor sampling interval. Hard real-time protection requires explanations with bounded latency, whereas maintenance-oriented explanations may be generated asynchronously after an alarm. Consequently, studies should report analysis-window length, update interval, inference latency, explanation latency, peak memory, and energy consumption separately.

The available evidence indicates that inherently interpretable lightweight models are generally more suitable for edge deployment than a black-box model followed by computationally intensive post hoc analysis. Nevertheless, parameter count is only a first-order indicator: memory is also determined by activation storage and numerical precision, while latency and energy depend on the target processor, sampling window, and software implementation. Quantization, pruning, and sensor-rate changes may additionally alter both predictions and explanations. Most reported models assume fixed preprocessing and hardware-independent signal quality, and only a small proportion provide measurements on actual microcontrollers or industrial edge devices. Claims of deployability and generalization should therefore be supported by hardware-specific memory, latency, energy, and explanation-stability tests, as summarized in Table 5.

Several strategies can reduce XAI overhead at the edge. First, inherently interpretable signal-processing or physics-guided models can generate diagnostic evidence during the forward pass without an additional explainer. Second, attribution can be calculated over physically meaningful frequency bands, orders, or sensor groups instead of individual samples. Third, perturbation-based methods can use reduced background sets, sparse sampling, or compact surrogate models. Fourth, explanation generation can be event-triggered, with lightweight detection performed continuously and detailed explanations generated only for alarms, confidence drops, or distribution shifts. Finally, a hierarchical architecture can retain time-critical detection on the embedded node while transferring more expensive explanation and historical comparison to an industrial gateway or supervisory server. These strategies reduce average latency but should still be evaluated for fidelity loss and explanation drift.

Table 3 summarizes algorithm-level explanation overhead, whereas Table 5 reports hardware-specific memory, latency, and energy evidence. Together, they distinguish theoretical computational suitability from hardware-verified real-time deployability and industrial validation.

#### 4.3.2. Explainable Diagnosis Methods for Strong Interference and Non-Stationary Conditions

The complex industrial environment characterized by strong background noise, variable rotational speeds, and varying loads not only leads to a sharp decline in diagnostic accuracy, but also causes post hoc explainability methods to output spurious explanations that fail to truthfully reflect the model’s decision-making logic. This constitutes a core challenge for the deployment of explainable diagnosis technology. Existing research has constructed a robust explainable diagnosis system under complex environments from three dimensions: front-end noise suppression, physical enhancement at the feature level, and optimization of explanation result robustness.

To address the difficulty of balancing model generalization and explainability under variable conditions, the causal filtering disentanglement domain generalization network incorporates a causal filter in the wavelet domain a priori. By suppressing non-causal noise and enhancing invariant causal features, the method maintains >92% accuracy at −4 dB SNR. Time-domain and envelope-spectrum visualizations trace the entire enhancement process, offering clear physical interpretability for model decisions under severe noise [114]. To meet the demand for robust diagnosis under strong noise, the two-layer multi-expert wavelet transformer designs in parallel an explainable multi-wavelet sparse representation denoising layer. It maintains a diagnostic accuracy of 88.32% under a −8 dB SNR. Through attention weight visualization and feature distribution analysis, stable traceability and reliable interpretation of the model’s decision-making logic under strong noise interference are realized [115]. The hyper-feature space graph collaborative embedding framework improves diagnostic robustness and the reliability of explanation results under strong noise from the perspective of graph-structured multi-source information aggregation [116]. The interpretable base learning autoencoder learns physically constrained basis features, maintaining a diagnostic accuracy of over 99% under a −8 dB noise environment. Through basis matrix visualization, full-process traceability of the decision-making logic is achieved [117].

Nevertheless, the evidence summarized above should primarily be regarded as laboratory robustness validation rather than direct industrial validation. Most studies simulate interference by adding Gaussian noise at predefined SNR levels or by changing speed and load within a controlled test rig. These procedures provide repeatable comparisons but cannot fully reproduce sensor degradation, installation variability, data-acquisition faults, electromagnetic interference, or long-term distribution shifts encountered in industrial plants. Therefore, high diagnostic accuracy under synthetic noise does not necessarily demonstrate reliable prediction or explanation under real operating conditions.

A recent bearing-diagnosis framework further combines a multiscale CNN, bidirectional multiscale gated recurrent units, and multi-head attention, with an improved whale optimization algorithm used for hyperparameter selection. Tests on public and experimentally collected datasets indicate improved diagnostic robustness under noise and variable operating conditions [118]. However, the stacked architecture and population-based optimization increase training complexity, and the attention module was primarily used to improve feature extraction rather than to establish explanation fidelity. Therefore, this study provides useful robustness evidence but should not be treated as conclusive evidence of explainability.

From a comparative perspective, front-end physics-guided denoising and invariant-feature learning are more likely to preserve explanation stability than applying a post hoc explainer after a noise-sensitive classifier has already made its decision. However, multi-stage denoising, recurrent modeling, graph aggregation, and population-based hyperparameter optimization increase training cost and may obscure which module produces the reported robustness. Moreover, most methods assume additive stationary noise and controlled speed or load variation; these assumptions do not cover electromagnetic interference, sensor faults, or long-term explanation drift. Robust generalization should therefore be demonstrated through cross-machine tests and repeated explanation-consistency measurements under realistic disturbances, not through classification accuracy at selected SNR levels alone.

#### 4.3.3. Explainable Diagnosis Methods for Data-Scarce Scenarios

In industrial scenarios, the collection of fault samples from rotating machinery is challenging, and datasets commonly suffer from class imbalance and small-sample or even zero-shot problems. Traditional models are prone to overfitting and low recognition rates for minority class faults. Due to insufficient feature learning, explainability methods fail to output reliable results. Existing research integrates explainability techniques with few-shot learning and imbalanced data processing, while one-shot transfer learning has also been explored to improve bearing diagnosis with extremely limited training samples [119].

To address heterogeneous class imbalance, the improved majority weighted minority oversampling technique combines hierarchical clustering, dynamic sampling, and intra-subcluster generation to reduce both intra- and inter-class imbalance. It outperformed mainstream oversampling methods across 20 FD cases, while cluster visualization made the process from sample generation to classification traceable [120]. For data-scarce scenarios, a decoupling GAN integrates CWT-based physical priors to separate intrinsic fault features from operating-condition interference and synthesize high-fidelity samples, improving zero-shot diagnostic accuracy for unknown fault sizes by over 20 percentage points [121]. Multi-view contrastive Shapelet learning extracts discriminative local patterns from limited labeled data and explains decisions through Shapelet matching and visualization [122]. A classifier-GAN-based semi-supervised framework jointly performs fault classification and sample-source discrimination, with channel-attention weights revealing its feature-learning process [123]. Finally, a mechanism-constrained decomposition diffusion network uses correlated kurtosis as a physical constraint, enabling compound-fault diagnosis from a single labeled sample and visualizing the decomposition and reconstruction process [124].

For cross-machine small-sample diagnosis, PCASTNet separates fault-related content from machine-specific style and introduces a band-energy preservation constraint based on physical frequency-domain knowledge. Its multiscale wavelet encoder and adaptive style-transfer mechanism generate target-machine fault samples while retaining fault-sensitive spectral energy, thereby improving diagnosis in two cross-machine scenarios [125]. Compared with unconstrained generative models, this approach provides a clearer physical basis for evaluating generated samples. Nevertheless, its effectiveness depends on the validity of the predefined frequency-band prior, and the interpretability of sample generation does not directly guarantee that the downstream classifier makes physically consistent decisions.

The main trade-off in data-scarce diagnosis is between explicit pattern transparency and flexible sample generation. Shapelet and physically defined feature approaches expose the local patterns supporting a prediction and are relatively economical, but they assume that a small number of recurring motifs sufficiently represent each fault. GAN, diffusion, and style-transfer methods can cover a broader sample space, yet their training cost is higher, and their generalization depends on the similarity between simulated, source-machine, and target-machine distributions. Physical constraints improve sample plausibility but cannot eliminate mode collapse, synthetic artifacts, or incorrect priors. Importantly, an interpretable generation process does not establish the fidelity of the downstream classifier. Evaluation should therefore separate sample quality, diagnostic performance, explanation stability, and cross-machine transfer rather than reporting accuracy improvement alone.

#### 4.3.4. Explainable Methods for Full Lifecycle Health Management

With the deepening adoption of the full lifecycle health management concept, intelligent FD of rotating machinery has expanded from single fault classification to the entire PHM chain, including early anomaly detection, performance degradation assessment, and RUL prediction. This imposes higher requirements on explainability, extending from fault identification interpretation to operational maintenance decision support. Existing research has deeply integrated explainability techniques into the full PHM chain, constructing a comprehensive explainable technology system covering anomaly detection, degradation assessment, and life prediction.

In RUL prediction, a deep transfer network based on dual-task learning employs collaborative learning of health status assessment and RUL prediction as dual tasks. Combined with physically constrained loss and transfer learning, it achieves high-precision life prediction across operating conditions. The model’s learning logic can be revealed through loss weight analysis [126]. The restraint graph attention network-reversible instance normalization transformer spatiotemporal graph attention framework intuitively displays the entire degradation process of bearings through spatiotemporal dynamic graph visualization. By introducing an expert-controllable constraint switch, it achieves observability and intervenability of the prediction process, significantly reducing prediction errors [127]. To address the explainability requirements of full lifecycle equipment health management, an explainable prediction method based on a binary pattern tracking health indicator and bidirectional gated recurrent unit is proposed, achieving explainable modeling of the full lifecycle degradation process of rolling bearings and accurate RUL prediction [128].

Recent time-series architectures also provide relevant technical foundations for full-lifecycle health management. The ZSTT model combines Transformer-based sequence modeling, approximate Bayesian inference, and dynamic pattern masking to perform zero-shot performance-degradation prediction for hydraulic actuators without target-domain pretraining [129]. A TCN-BiLSTM model similarly combines dilated causal convolution with bidirectional temporal modeling for predicting the occurrence time of industrial equipment faults [130]. These models illustrate advances in long-term dependency modeling, cross-system generalization, and uncertainty-aware prediction. However, neither model directly explains its predictions in terms of rotating-machinery fault mechanisms; therefore, they are included as adjacent lifecycle methods rather than direct evidence of explainable fault diagnosis.

In early anomaly detection and performance degradation assessment, a method based on subspace-whitening support vector data description achieves refined and explainable assessment of diesel engine performance degradation through multi-source impulse signal decomposition and joint feature subspace construction, enabling independent quantification of the degradation status of each component [131]. A sequence holospectrum conditional probability density autoencoder deeply integrates rotor dynamics holospectrum technology with DL, achieving high-sensitivity early warning of early faults through temporal probabilistic evolution analysis of holospectrum parameters [132]. A multi-granularity scanning extended isolation forest with an adaptive threshold strategy achieves fast and explainable unsupervised anomaly detection for rotating machinery. The inherent logic and decision-making basis of anomaly detection can be clearly explained through feature separation visualization, anomaly score distribution analysis, and path length statistics [133].

Furthermore, for cross-device and cross-condition generalized FD of bearing-rotor systems, relevant studies have systematically revealed the inherent mechanisms of cross-domain feature alignment and knowledge transfer through methods such as quantitative modeling of distribution differences and low-dimensional embedding feature visualization [134,135]. For multi-sensor multi-modal fusion diagnosis solutions, the transparency of the multi-source information fusion process is achieved through graph relationship evolution analysis, further expanding the industrial application boundaries of explainability techniques [136].

For multi-sensor diagnosis, Wang et al. combined heuristic information gain with ParallelGraphNet to select informative sensor locations and jointly extract spatial and temporal features. Validation using a high-speed elevator platform and a public gearbox dataset demonstrated that sensor selection can reduce redundant acquisition while maintaining diagnostic performance [137]. The selected sensor set provides engineers with an understandable indication of measurement relevance, but information-gain ranking does not explain how individual signal characteristics determine the final fault class. Decision-level XAI validation is therefore still required.

Full-lifecycle methods must explain temporal evolution rather than only a final fault class. Health-indicator and mechanism-based approaches provide clearer degradation trajectories, whereas attention-based recurrent or Transformer models capture long-range dependencies but require more computation and may offer only correlational explanations. Cross-condition transfer and multi-sensor fusion improve coverage, but they assume comparable degradation stages, sensor semantics, and maintenance definitions across assets. Generalization is therefore more demanding than in fault classification, because errors accumulate over long prediction horizons and maintenance actions depend on calibrated uncertainty. Practical systems should report explanation consistency across degradation stages, prediction intervals, update latency, and whether the explanation changes an operator’s maintenance decision, rather than relying solely on RUL error or feature visualization.

## 5. Research Challenges and Future Directions

Systematic review shows explainable IFD for rotating machinery has matured from theory to lab validation, with initial industrial deployment explorations underway. However, there are still a series of core bottlenecks and unresolved key challenges in promoting the large-scale industrial deployment of these technologies. This section systematically sorts out the core challenges facing the current research, and on this basis, discusses the key future research directions oriented to industrial deployment requirements.

### 5.1. Core Challenges Facing Current Research

Currently, a quantitative evaluation system for explainability remains absent, and a unified industry benchmark has yet to be established. Most validation of explainability relies on qualitative visualization techniques, lacking recognized quantitative metrics for transparency, comprehensibility, and credibility. There is also a shortage of standardized test datasets. This makes it difficult to conduct fair horizontal comparisons among different methods, thereby constraining the standardization and promotion of related technologies.

The inherent contradiction between diagnostic performance and explainability has not yet been fundamentally resolved. Ante hoc mechanism embedding methods possess native explainability, but the physical constraints limit the nonlinear representation capacity of the models, making it hard for their accuracy to match that of high-performance models in complex scenarios. Although post hoc explainability methods preserve model accuracy, their explanation results are merely approximate reconstructions of the decision-making logic, carrying the risk of spurious explanations.

Under complex industrial scenarios, the robustness and adaptability of explainability methods remain insufficient. Faced with stringent conditions such as strong on-site noise, variable operating conditions, limited edge computing power, and scarce fault samples, issues arise including explanation result drift, loss of explainability during model lightweighting, and explanation results lacking physical meaning in small-sample settings.

The integration between explainability and industrial O&M decision-making is still shallow, making it difficult to effectively realize engineering value. Existing research mainly focuses on improving explainability at the algorithmic level, with output results remaining at the level of feature-focused regions. They have not been translated into decision-making information directly applicable to on-site maintenance, such as fault localization, root cause analysis, and maintenance strategy optimization, resulting in a clear disconnect from actual industrial requirements.

### 5.2. Laboratory-to-Industrial Validation Gap

A major limitation of current explainable fault-diagnosis research is the predominance of laboratory validation. Public datasets and test-rig experiments generally involve seeded faults, controlled sensor installation, limited speed and load combinations, relatively balanced classes, and short acquisition periods. In contrast, industrial machinery is affected by naturally evolving incipient or compound faults, machine-to-machine differences, maintenance interventions, continuously changing operating conditions, and severely imbalanced fault records. Recent reviews have similarly identified imperfect data, distribution shift, and insufficient field validation as major barriers to transferring intelligent diagnosis models from theoretical studies to industrial applications [138,139].

Sensor quality represents an additional but insufficiently investigated source of uncertainty. Low-cost, aging, or improperly installed sensors may exhibit limited bandwidth, a high noise floor, reduced dynamic range, saturation, clipping, sensitivity drift, temperature-dependent bias, loose mounting, sampling jitter, and intermittent data loss. These problems may suppress genuine fault components or introduce artificial spectral peaks, potentially causing both incorrect predictions and misleading XAI attributions. Research on vibration-based monitoring has demonstrated that sensor faults can produce false alarms and distort condition-assessment results [140]. Future studies should therefore report sensor type, sensitivity, bandwidth, mounting position, calibration status, sampling configuration, and missing-data handling, and should evaluate explanation stability under controlled sensor degradation.

Electromagnetic interference differs fundamentally from the stationary Gaussian noise commonly used in laboratory robustness tests. Variable-frequency drives, pulse-width-modulated power converters, motors, power cables, and grounding loops can generate conducted or radiated interference with non-stationary, impulsive, and harmonic characteristics. Such interference may contaminate fault-related frequency bands and introduce spurious peaks into envelope spectra. Kim et al. demonstrated that EMI generated by variable-frequency drives can corrupt bearing condition-monitoring signals and obscure diagnostically relevant spectral components [141]. Consequently, adding white Gaussian noise at a specified SNR cannot be considered sufficient evidence of EMI robustness.

Industrial validation should evaluate diagnostic performance and explanation stability separately. In addition to accuracy, the consistency of explanations before and after sensor degradation or EMI exposure should be quantified using rank correlation, cosine similarity, top-k feature overlap, or localization agreement. A progressive validation protocol is recommended, beginning with public benchmark datasets, followed by cross-rig and cross-machine experiments, tests using naturally degraded or imperfect sensor data, and prospective on-site validation. EMI tests should also distinguish radiated, conducted, and transient interference rather than representing all disturbances using a single additive-noise model.

### 5.3. Integration of XAI Outputs with Industrial Vibration-Diagnosis Standards

Although XAI methods can visualize the time segments, frequency components, or input features that influence a diagnostic decision, these outputs cannot be directly regarded as engineering measurements. Attribution scores generated by SHAP, Grad-CAM, integrated gradients, or attention mechanisms are model-relative and generally dimensionless. Moreover, explanation fidelity only indicates whether an explanation reflects the prediction logic of the model; it does not demonstrate that either the model or its explanation is physically correct. Therefore, XAI outputs should be converted into physically meaningful and verifiable diagnostic evidence before being used to support industrial maintenance decisions [142].

For industrial application, XAI outputs should be interpreted within a standardized vibration-measurement and diagnostic chain. ISO 13373-1 specifies general requirements for sensor selection, sensor location, transducer attachment, operating conditions, and vibration data collection [143]. ISO 16063-21 provides procedures for calibrating vibration transducers by comparison with a reference transducer [144]. The acquired signals can then be processed and presented using the time-domain, frequency-domain, order-domain, and envelope-spectrum procedures recommended in ISO 13373-2 [145]. ISO 13373-3 provides general guidelines for relating vibration signatures to possible machine faults [146]. Vibration magnitude and severity should be reported according to ISO 20816-1 and applicable machine-specific parts, such as ISO 20816-3 [147,148]. These standards do not prescribe particular XAI algorithms; the mapping between XAI outputs and standardized vibration evidence proposed in Table 6 is therefore an analytical framework developed in this review.

The mapping between XAI outputs and industrial vibration standards is proposed by this review. The cited ISO standards define measurement, processing, diagnosis, and severity-evaluation requirements, but do not specify XAI metrics or explanation algorithms.

As shown in Table 6, XAI outputs should supplement rather than replace calibrated vibration measurements, physical fault evidence, and machine-specific severity criteria.

XAI outputs can be translated into practical engineering evidence by mapping attribution results onto these standardized signal representations. For example, in bearing diagnosis, shaft speed and bearing geometry can first be used to calculate the ball-pass frequency of the outer race, ball-pass frequency of the inner race, ball-spin frequency, and fundamental train frequency. Attribution values can then be aggregated within tolerance bands around these frequencies, their harmonics, and modulation sidebands. The resulting attribution should be reported together with the corresponding acceleration, velocity, spectral amplitude, envelope energy, or trend value in physical units. In this way, an abstract heatmap can be converted into an engineering statement, such as “the diagnostic decision is primarily supported by an increasing envelope component around the outer-race fault frequency and its second harmonic.”

The engineering meanings of different XAI metrics should also be clearly distinguished. Fidelity indicates whether an explanation represents the model decision, whereas robustness and stability describe whether similar signal segments produce repeatable explanations. Localization can be evaluated according to the overlap between highlighted regions and physically expected fault-frequency bands, while physical consistency reflects agreement with machine kinematics and known fault mechanisms. Sparsity determines whether the explanation can be reduced to a manageable number of diagnostic features. When measurement validity, explanation stability, or physical consistency is insufficient, the diagnostic result should be referred for manual inspection rather than being used to trigger an automatic maintenance action.

Taken together, Table 3, Table 4, Table 5 and Table 6 establish a progressive analytical sequence from methodological comparison and method selection, through deployment feasibility, to standard-aligned engineering interpretation and maintenance decision support.

### 5.4. From Correlational Explanation to Causal Root-Cause Analysis

Most post hoc XAI methods explain statistical associations between model inputs and predictions rather than causal relationships among machine components. A high attribution assigned to a frequency band indicates that the model relies on that signal component, but it does not establish whether the corresponding component initiated the fault. In a coupled motor–shaft–gearbox system, for example, a gearbox defect may generate secondary responses in the motor current, shaft vibration, and adjacent bearing signals. SHAP, Integrated Gradients, or CAM may highlight all these responses without identifying their temporal or physical direction. By contrast, Causal AI represents component states and their dependencies through a directed causal graph and distinguishes observational association from intervention and counterfactual reasoning. Within an SCM, the root cause is the upstream variable whose intervention changes or removes the downstream abnormal responses, rather than simply the variable receiving the highest attribution score [149,150].

A causal IFD framework can be constructed by representing component health states, operating conditions, transmission paths, signal features, and diagnostic outcomes as nodes in a directed graph. Initial edges can be defined from machine topology, rotor dynamics, fault-characteristic frequencies, and expert knowledge, and subsequently refined using temporal causal discovery from multi-sensor data. Conventional XAI can first identify candidate signal regions or sensor channels associated with a diagnosis. SCM-based intervention can then estimate whether setting a candidate component to a healthy state would suppress abnormalities in downstream variables, while counterfactual inference can predict how the observed signals would have evolved in the absence of that component fault. Digital twins and controlled fault-injection experiments can provide additional interventional evidence when field interventions are unsafe or unavailable.

Recent causal representation and domain-generalization methods for rotating machinery aim to separate fault-related invariant features from condition-dependent correlations, thereby improving diagnosis under unseen operating conditions [151,152]. However, causal filtering, causal disentanglement, or attention-based feature selection should not automatically be regarded as causal root-cause analysis. For example, CFDNet improves the extraction of invariant diagnostic features but does not by itself establish an explicit component-level SCM or verify fault-propagation direction. A credible causal diagnosis framework should therefore report the assumed causal graph, distinguish expert-defined from data-discovered edges, quantify uncertainty in causal direction, and validate root-cause and propagation-path identification using known fault initiation, controlled interventions, or counterfactual recovery tests. Without such validation, causal terminology may merely relabel robust correlation learning.

### 5.5. Key Future Research Directions

Future research should first establish standardized evaluation frameworks for explainable rotating machinery fault diagnosis. The three dimensions proposed in this review—transparency, comprehensibility, and credibility—should be operationalized through metrics such as fidelity, robustness, stability, localization, physical consistency, computational overhead, and human interpretability. Benchmark datasets and reporting protocols should cover different machinery types, fault modes, operating conditions, naturally evolved faults, sensor imperfections, and representative industrial disturbances. This would enable reproducible comparison beyond diagnostic accuracy alone.

Second, explainable diagnosis should progress from correlation-based visualization toward physics-guided and causally grounded reasoning. Fault dynamics, signal-processing principles, component relationships, and uncertainty information should be integrated into model architectures, causal inference frameworks, and digital twins. Existing digital-twin research has demonstrated the potential to connect equipment-state synchronization, fault diagnosis, visualization, and operator interaction within a unified decision-support platform [153]. Physical-data-driven closed-loop frameworks further show how model evaluation, error correction, and feedback optimization can support industrial decision-making [154]. Future digital twins for rotating machinery should additionally link XAI outputs with fault-characteristic frequencies, explanation uncertainty, vibration standards, and verified maintenance actions.

Third, explainability, robustness, and computational efficiency should be jointly optimized for industrial deployment. Future systems should be evaluated under sensor degradation, electromagnetic interference, variable speed and load, cross-machine distribution shifts, limited fault samples, and edge-computing constraints. Lightweight models should report hardware-specific memory, latency, and energy consumption, while explanation stability and drift should be monitored together with diagnostic performance. The close coupling among control, fault diagnosis, and fault-tolerant operation in mobile machinery also highlights the need for coordinated decision support across the equipment life cycle [155].

Finally, human-centered explainability should be treated as a socio-technical requirement rather than a visualization add-on. Maintenance technicians generally require concise information about fault location, severity, confidence, supporting evidence, and recommended action, whereas engineers may need detailed waveforms, spectra, time-frequency maps, and attribution results. Human-centered XAI should therefore be evaluated through user understanding, usability, trust calibration, workload, decision time, and human–AI collaboration performance [156]. A progressive-disclosure interface can provide an actionable alarm layer, a standardized evidence layer, and a detailed expert-analysis layer. Prediction confidence, explanation stability, sensor quality, operating-condition warnings, and unresolved uncertainty should be displayed to reduce automation bias. When these indicators are insufficient, the system should require human confirmation or escalation rather than issuing an autonomous maintenance command [157].

## 6. Conclusions

This comprehensive review examined ante hoc and post hoc XAI methods for rotating machinery fault diagnosis from methodological and industrial perspectives. Ante hoc methods provide stronger physical traceability by embedding signal-processing operators, mechanism knowledge, or transparent structures, but their effectiveness depends on the validity of the incorporated prior knowledge. Post hoc methods can explain existing black-box models more flexibly, although their outputs may be unstable, computationally demanding, and correlational rather than causal. The comparative analysis demonstrates that diagnostic accuracy alone is insufficient for evaluating explainable diagnostic models. Explanation fidelity, robustness, stability, localization, physical consistency, computational overhead, memory consumption, and hardware-specific latency should also be assessed.

Current evidence remains dominated by seeded faults, public datasets, synthetic noise, and controlled operating conditions. Sensor degradation, electromagnetic interference, cross-machine distribution shifts, and long-term explanation drift therefore remain major barriers to industrial deployment. Industrial XAI should translate model-relative outputs into calibrated vibration quantities, fault-characteristic frequencies, standards-aligned diagnostic evidence, and actionable maintenance recommendations, while supplementing rather than replacing physical measurements and expert judgment. Future research should prioritize standardized benchmarks, lightweight and robust explanation methods, causal root-cause reasoning, digital-twin integration, and human-in-the-loop validation. These developments are necessary to advance explainable fault diagnosis from laboratory visualization toward trustworthy industrial decision support.

## Figures and Tables

**Table 1:** Database-specific search settings and records retrieved.

Database	Search Field	Time Coverage	Language/Type Filters	Records
Web of Science	Topic/title/abstract/keywords	Inception–1 June 2026	English; article/review	918
IEEE Xplore	Title/abstract/metadata	Inception–1 June 2026	English; journal article/review	354
ScienceDirect	Title/abstract/author keywords	Inception–1 June 2026	English; research/review article	320

**Table 2:** Literature relevance criteria used in this comprehensive review.

Criterion	Included	Excluded
Machinery	Clearly defined rotating machinery or component	Non-rotating or undefined equipment
Task	Detection, classification, localization, or severity assessment	Unrelated tasks
XAI content	Explicit ante hoc or post hoc method	Black-box diagnosis without explanation
Evidence	Explanation result, attribution, localization, physical interpretation, or metric	Interpretability claimed without evidence
Publication	English peer-reviewed research/review article	Conference paper, thesis, patent, book, or non-peer-reviewed work
Reporting	Sufficient method and experiment information	Insufficient methodological information

**Table 3:** Method-level comparison of XAI approaches for rotating machinery fault diagnosis.

Method	Type	Best Use	Cost	Explanation Strength	Drift Robustness	Deployment Potential	Key Limitation
Signal-processing embedding	Ante hoc	Weak/periodic faults; noisy signals	Low–moderate	Strong	Moderate	High	Basis/band dependence
Physics-guided models	Ante hoc	Known mechanisms; scarce labels	Moderate–high	Strong	Moderate–high	Medium	Prior/model mismatch
Attention mechanisms	Ante hoc	Multi-sensor; long sequences	Low–moderate	Conditional	Low–moderate	Medium–high	Weights may be unfaithful
CAM/Grad-CAM	Post hoc	CNN time-frequency localization	Low–moderate	Approximate	Low	Medium	Coarse, unstable saliency
IG/LRP/DeepLIFT	Post hoc	Deep-model sample attribution	Moderate	Conditional	Low–moderate	Medium	Baseline/rule sensitivity
LIME	Post hoc	Model-agnostic local audit	High	Approximate	Low	Low	Sampling instability
SHAP	Post hoc	Global/local feature ranking	High	Conditional	Low–moderate	Low	Feature dependence; high cost

Note: Cost denotes the additional computation required to generate an explanation beyond the original diagnostic inference. Low indicates a single additional mapping or propagation stage; moderate indicates multiple gradient passes, activation storage, or additional embedded modules; and high indicates repeated perturbation, sampling, and model evaluation. Actual runtime depends on input length, model architecture, hardware platform, and software implementation.

**Table 4:** Quantitative metrics for evaluating explanation quality in rotating machinery fault diagnosis.

Metric	Operational Measure	Attribute	FD Evidence	Direction
Fidelity	Top-k deletion/insertion	Credibility	Segments, bands, channels	↑
Robustness	Similarity after disturbances	Credibility	Noise, speed, load changes	↑
Stability	Similarity across neighbors/runs	Credibility	Same-fault samples or seeds	↑
Sensitivity	Response to small perturbations	Credibility	Signal or model changes	Context-dependent
Monotonicity	Confidence drop after ranked removal	Transparency	Ranked features or bands	↑
Localization	Overlap with known fault regions	Comprehensibility	Fault bands or impulses	↑
Sparsity	Number/entropy of salient features	Comprehensibility	Features, bands, sensors	↓ *
Physical consistency	Agreement with fault mechanisms	Credibility	Orders, harmonics, sidebands	↑

↑: higher is preferred; ↓: lower is preferred. * Sparsity should be improved only when adequate fidelity is retained.

**Table 5:** Deployment and industrial-validation evidence of representative interpretable diagnosis models.

Ref.	Machinery/Data Source	Fault Origin	Disturbance Considered	XAI Mechanism	Hardware	Memory	Latency	Energy	Deployment Evidence
[48]	Three rotating-machinery weak/compound-fault datasets	Experimental; NR	Weak faults and noise	Random wavelet kernels	CPU/GPU comparison only	NR	NR; relative speed reported	NR	Laboratory validation
[51]	CWRU and HIT bearing datasets	Seeded/experimental	Additive noise down to −15 dB	Embedded wavelet transform	NR	NR	NR	NR	Laboratory validation
[56]	Aviation bearing, planetary gearbox and aero-engine data	Mixed; partly real-world	Noise and varying conditions	Spectral selection and graph-feature visualization	NR	NR; 0.083 M parameters	NR	NR	Industrial-data validation; no edge hardware
[63]	Self-collected gearbox and UO bearing datasets	Experimental; NR	Variable-speed conditions	Speed-guided FIR filters and prior indicators	NR	NR	NR	NR	Laboratory validation
[110]	Public rolling-bearing datasets	Experimental; NR	Additive-noise tests	S-GAP and interpretable convolution features	NR	NR; 310 parameters	NR	NR	Deployment potential only
[112]	Gear datasets and actual edge diagnosis test	Experimental; NR	Cross-condition/generalization tests	Sparse pulse kernel and subtraction convolution	Edge diagnosis node prototype	54 KiB	0.045 s	6.67 mJ	Actual edge-hardware validation
[114]	Four rotating-machine datasets	Experimental; NR	Unseen operating conditions and additive noise	Causal wavelet filtering and feature visualization	NR	NR	NR	NR	Cross-condition laboratory validation
[115]	Four rotating-machinery datasets	Experimental; NR	Additive noise down to −8 dB	Wavelet denoising and attention visualization	NR	NR	NR	NR	Lightweight potential; no hardware validation

**Table 6:** Mapping XAI outputs and evaluation metrics to industrial vibration-diagnosis evidence and maintenance decisions.

Engineering Stage	Standard Basis	XAI Output or Metric	Required Translation	Practical Engineering Insight
Measurement reliability	ISO 13373-1; ISO 16063-21	Attribution uncertainty and stability	Associate explanations with sensor location, calibration status, operating condition and physical units	Accept, reacquire or reject the measurement
Signal processing	ISO 13373-2	Time-, frequency- or order-domain attribution	Aggregate attribution over waveform segments, spectral bands, orders or envelope components	Identify the signal components supporting the decision
Fault diagnosis	ISO 13373-3	Fidelity, localization and physical consistency	Map highlighted regions to characteristic frequencies, harmonics, sidebands and machine components	Determine the probable fault type, location and mechanism
Severity evaluation	ISO 20816-1 and applicable machine-specific parts	XAI evidence combined with vibration magnitude and trend	Report attribution together with standardized vibration values and changes over time	Determine alarm severity and inspection urgency
Maintenance decision	ISO 13373-3 and plant-specific rules	Confidence, robustness and sparsity	Present the main evidence, explanation reliability and unresolved uncertainty	Inspect, lubricate, align, balance, replace or request expert review
Human review	ISO 13373-3; plant SOPs	Confidence, uncertainty, stability, workload	Present concise evidence and require confirmation when reliability is low	Accept, override, or escalate the maintenance recommendation

## Data Availability

The data presented in this study are available on request from the corresponding author.
